# Lineage Plasticity and Stemness Phenotypes in Prostate Cancer: Harnessing the Power of Integrated “Omics” Approaches to Explore Measurable Metrics

**DOI:** 10.3390/cancers15174357

**Published:** 2023-09-01

**Authors:** Souzana Logotheti, Eugenia Papadaki, Vasiliki Zolota, Christopher Logothetis, Aristidis G. Vrahatis, Rama Soundararajan, Vasiliki Tzelepi

**Affiliations:** 1 Department of Pathology, University of Patras, 26504 Patras, Greece; slogotheti@upnet.gr (S.L.); epapadaki@ionio.gr (E.P.); zol@med.upatras.gr (V.Z.); 2 Department of Informatics, Ionian University, 49100 Corfu, Greece; aris.vrahatis@ionio.gr; 3 Department of Genitourinary Medical Oncology, The University of Texas MD Anderson Cancer Center, Houston, TX 77030, USA; clogothe@mdanderson.org; 4 Department of Translational Molecular Pathology, The University of Texas MD Anderson Cancer Center, Houston, TX 77030, USA

**Keywords:** prostate cancer, tumor stemness, measuring lineage plasticity, NGS, genomic, transcriptomic, epigenetic alterations, bioinformatics

## Abstract

**Simple Summary:**

Prostate cancer remains the most frequent cause of cancer morbidity, the second most frequent cause of cancer mortality in men in the developed world and is an exemplar of a heterogeneous disease. Stemness phenotypes and lineage plasticity have been highlighted as key drivers of heterogeneity observed both across patients and within the same patient. However, markers that indicate the presence or absence of these events remain to be identified. Next-generation sequencing has proven to be a beneficial approach to distinguish predictive and prognostic biomarkers in various diseases, including prostate cancer. This review explores measurable metrics that can reliably reflect lineage plasticity at the genomic, transcriptomic, and epigenomic levels, as well as bioinformatic tools that can be used to identify measures of lineage-plasticity in prostate cancer, in order to inform preclinical and clinical research.

**Abstract:**

Prostate cancer (PCa), the most frequent and second most lethal cancer type in men in developed countries, is a highly heterogeneous disease. PCa heterogeneity, therapy resistance, stemness, and lethal progression have been attributed to lineage plasticity, which refers to the ability of neoplastic cells to undergo phenotypic changes under microenvironmental pressures by switching between developmental cell states. What remains to be elucidated is how to identify measurements of lineage plasticity, how to implement them to inform preclinical and clinical research, and, further, how to classify patients and inform therapeutic strategies in the clinic. Recent research has highlighted the crucial role of next-generation sequencing technologies in identifying potential biomarkers associated with lineage plasticity. Here, we review the genomic, transcriptomic, and epigenetic events that have been described in PCa and highlight those with significance for lineage plasticity. We further focus on their relevance in PCa research and their benefits in PCa patient classification. Finally, we explore ways in which bioinformatic analyses can be used to determine lineage plasticity based on large omics analyses and algorithms that can shed light on upstream and downstream events. Most importantly, an integrated multiomics approach may soon allow for the identification of a lineage plasticity signature, which would revolutionize the molecular classification of PCa patients.

## 1. Introduction

Prostate cancer (PCa) remains the most frequent and second most lethal cancer in men in the developed world [1], and is an exemplar of heterogeneous disease. According to the American Cancer Society, over 280,000 new cases of PCa and over 34,000 deaths due to this disease are anticipated in 2023 in the United States. The clinical course of PCa varies from indolent behavior (which requires minimal, if any, therapeutic intervention) to aggressive disease that progresses rapidly and is resistant to therapy. Morphologically, inter- and intratumor heterogeneity has been observed in PCa. The latter is best described with the Gleason score, which represents the sum of the two most prevalent histologic patterns, with tertiary patterns frequently present and separately accounted for in the pathology report [2,3].

Therapeutic options vary depending on the disease stage, Gleason score, and serum PSA levels, as well as patient age, comorbidities, and preferences [4]. In most cases, PCa is an AR-dependent tumor; thus, androgen deprivation therapy (ADT) and AR signaling inhibition (ARSI) therapy persist as the mainstay systemic therapies for patients with recurrent or metastatic PCa [5,6,7]. While most patients have a long-term response to ADT, many cancers do recur, leading to castrate-resistant prostate cancer (CRPC). While the majority of CRPC tumors remain AR-driven through various mechanisms, including the acquisition of activating *AR* mutations, *AR* gene amplifications, ligand-independent *AR* splice variants, or ligand promiscuity, up to 20% of CRPC tumors adapt to or lose AR dependence as a means to evade *AR*-targeted therapy. In these patients, aggressive, atypical clinical features ensue, including lytic bone metastases, visceral dissemination, and low PSA levels for disease burden. Histologically, a transformation from adenocarcinoma to small-cell (neuroendocrine) PCa has been seen in this setting in some, but not all, cases [8,9]. The term aggressive variant prostate cancer (AVPC) is used to describe this disease state, which has limited therapeutic options and accounts for 30% of PCa deaths [10,11]. Many clinical and preclinical efforts have been undertaken to elucidate the underlying biology of disease evolution toward the *AR*-indifferent cell state in order to identify biomarkers that could facilitate the early recognition of these patients, as well as potential therapeutic targets.

Lineage plasticity, defined as the ability of cells to change their differentiation state, has emerged as a significant hallmark of cancer progression and treatment resistance, and has been proposed as a source of intratumoral heterogeneity [12,13,14]. Based on this ability, neoplastic cells can adapt by switching from one committed developmental pathway to another, and this transformation has been proposed as a driver of intratumoral heterogeneity and cancer progression [12]. The best-studied phenotype of cancer lineage plasticity is the epithelial-to-mesenchymal transition (EMT), which allows neoplastic cells to transform to a less differentiated state with enhanced tumorigenic and metastatic properties [15,16,17]. In EMT, the epithelial phenotype of the cell changes dynamically toward a mesenchymal phenotype, while the tumor progresses in order to bypass either immunologic or therapeutic barriers [18]. Similarly, lineage plasticity has been implicated in the development of aggressive and treatment-resistant phenotypes, such as neuroendocrine and small-cell PCa [19]. It has also been hypothesized to account for the phenotypic heterogeneity of AVPCs and the bidirectional transition of cancer cells between two morphologic and molecular states: AR-driven adenocarcinoma cells and AR-indifferent cells of small-cell and various other morphologies [20]. Hence, lineage plasticity may be used as an additional classifier for patients with PCa, with patients belonging to one of two groups: a group with lineage-constrained disease, which includes patients who respond well to current treatment strategies and whose cancer may expand with consistent histology or slow phenotypic changes and better prognosis [21,22,23,24], and a group with lineage-plastic disease, which includes patients with short or no response to current therapies, whose cancer progresses rapidly with diverse histology and poor outcomes [10,19,25,26,27,28,29]. Such classification would represent a fundamental milestone, enabling clinicians to predict the response to AR inhibition, identify aggressive variants, and facilitate the emergence of new therapeutic targets. However, lineage plasticity measures remain to be defined.

In routine clinicopathological evaluation, histology (morphology and Gleason score/grade group), imaging, and clinical TNM staging are applied to characterize PCa heterogeneity and identify potential lineage plasticity from a histological/clinical perspective [30,31,32,33]. These methods provide valuable information about the tumor heterogeneity, differentiation status, and aggressiveness, which can help researchers and clinicians better understand the clinical implications of lineage plasticity in cancer. However, finer molecular characterizations of tumor heterogeneity and lineage plasticity are needed. In recent years, research has increasingly shifted toward molecular insights driven by next-generation sequencing (NGS) technologies [34]. These advanced methods offer significant opportunities for the in-depth analysis of lineage plasticity in cancer. In particular, single-cell technologies, such as single-cell RNA sequencing (scRNA-seq), enable researchers to perform detailed analyses of tumors at the individual cell level, offering a more profound understanding of cellular heterogeneity and lineage plasticity.

Lineage plasticity is reported to be enabled through genomic and epigenetic events [35], encompassing two differentiation scenarios: (a) dedifferentiation, which refers to a transition from a fully differentiated cell state to a less differentiated cell state, and (b) transdifferentiation, which refers to a transition from a fully differentiated cell state to an alternative fully differentiated cell state [36]. In PCa, lineage plasticity tracing studies have demonstrated that neuroendocrine prostate cancer (NEPC) cells originate from luminal cells in response to ADT, supporting the transdifferentiation scenario [37]. While genetic alterations, such as *ETS* (E26 transformation-specific) gene fusions, have been identified as drivers of PCa initiation and progression, recent evidence has highlighted the importance of epigenetic and transcriptomic alterations in promoting lineage plasticity [27,38]. As bidirectional inherited changes in chromatin structures that modify gene expression and cell phenotype without any genomic changes, epigenetic alterations represent an ideal mechanism for the development of lineage plasticity [39,40]. Indeed, DNA methylation and histone modifications have been shown to alter gene expression programs and promote lineage plasticity [41]. Similarly, changes in transcription factor expression and activity can promote lineage plasticity by driving cells toward alternative differentiation states [42]. Therefore, epigenetic changes can enhance the switch between different developmental states in accordance with the microenvironmental pressures that occur under various therapeutic strategies.

Cancer stem cells (CSCs) have been proposed as key factors accounting for intratumoral heterogeneity, tumor progression, and evolution [43]. However, the more specific transformation of a neoplastic cell to a stem-like state is a plasticity event that may reveal the true driver in specific contexts [35,44]. Lineage plasticity is associated with the stem-like behavior of neoplastic cells. While stem cells can give rise to different developmental cell states, lineage-plastic cells adapt to environmental changes for the sake of their survival by switching between different cellular conditions. A recent study identified a mesenchymal and stem-like PCa cell state as a result of an ARSI-therapy-induced lineage plasticity response [45]. A detailed investigation into relevant molecular and epigenetic factors, as well as the identification of a cell population that shows stem-like or lineage plasticity-like characteristics, would help elucidate the underlying biology of tumor progression, tumor heterogeneity, therapy resistance, and metastasis.

In PCa, the Yamanaka pluripotent factor *SOX2* has been associated with aggressive disease [27,46,47]. The *FOXC2* protein has also been identified as a candidate stem cell marker in aggressive NEPC. The gain of function of this marker has been linked to therapy resistance (enzalutamide and docetaxel) and the epithelial-to-neuroendocrine transdifferentiation, while the loss of function has been shown to restore ADT sensitivity and the neuroendocrine-to-epithelial transformation both in vitro and in vivo [48]. Various stem cell-associated signaling cascades, including EMT and EMT-related pathways (TGF-β pathway, Wnt signaling, and Hedgehog pathway), PI3K-AKT-mTOR signaling, JAK/STAT, and others, have been reported to play key roles in the development of aggressive PCa phenotypes and are potentially linked to lineage plasticity [49,50]. EMT and mesenchymal-to-epithelial transition (MET) pathways enable neoplastic cells to transform from an epithelial to a mesenchymal morphology, and metastasize from the site of origin to distant sites returning to an epithelial morphology [51,52,53]. These pathways are best described as “flavors” of lineage plasticity that have been able to elucidate how neoplastic cells metastasize and grow in distant sites. In addition, the N-Myc and Aurora kinase-A pathways have been shown to be upregulated in aggressive PCa phenotypes and have been suggested as candidate pathways that fuel lineage plasticity events, potentially serving as targets for therapeutic strategies [54,55,56,57,58,59,60].

In recent years, “omics” has revolutionized cancer research and treatment by enabling a more comprehensive understanding of the molecular complexity underlying cancer development and progression. Approaches such as genomic, transcriptomic, and epigenomic analyses have showcased the potential to decipher the intricate genetic and epigenetic alterations and molecular pathways that drive tumorigenesis [61], including the identification of key oncogenic drivers and candidate therapeutic targets towards facilitating the development of personalized medicine approaches [62]. As the field continues to evolve, the integration of omics data into clinical practice holds tremendous promise in transforming cancer care and, ultimately, increasing the chances of achieving successful cancer management.

In this review, we discuss the current research advances in the evaluation of lineage plasticity in PCa, including genomic and epigenetic data in support of PCa evolution and progression through various plastic cell states. We discuss the need to identify a lineage plasticity signature using transcriptomic and epigenomic techniques, and address candidate measures that have been studied for other types of cancer that could potentially be leveraged in PCa. We further discuss bioinformatic tools that could be employed in the development of lineage plasticity signatures.

## 2. Genomic Drivers of PCa Progression and Evolution

Significant advancements in genomic technologies have enabled researchers uncover the genetic landscape of PCa. Genome-wide association studies have identified numerous single-nucleotide polymorphisms (SNPs) associated with an increased risk of developing PCa, while NGS has revealed a diverse array of somatic genomic alterations present in PCa, including point mutations, gene fusions, copy number alterations, and structural variations [63]. Table 1 summarizes the most frequent genomic events observed in prostate cancer. Here, we describe these genetic events and their role in prostate cancer development, progression, and evolution, and we highlight the events that correlate with lineage plasticity in PCa.

Gene fusions of androgen-regulated genes and members of the *ETS* family of transcription factors are the most common genetic events in primary PCa, with radical prostatectomy and biopsy specimens showing ETS fusions in 27% to 79% of cases [65,66]. In addition, mutations in the *SPOP* gene, which encodes an E3 ubiquitin ligase, are the second most common genetic event and the most common mutation in PCa. *SPOP* mutations have been associated with alterations in AR signaling and DNA damage repair pathways, as well as resistance to *BET* inhibitors through the stabilization of *BRD4* [82,83,84,85]. *SPOP* mutations and *ETS* fusions are mutually exclusive, and studies have identified molecular subtypes of PCa based on the genetic driver present; however, these genetic events have not conferred any prognostic or predictive information to date, nor could they be used to guide therapy selection.

Secondary genetic events include the loss of the *PTEN* tumor suppressor gene, which is located on chromosome 10. *PTEN* loss is present in approximately 15% to 20% of primary PCa cases and in 40% to 60% of cases upon disease progression, with greater frequency in ETS-rearranged cases, and has been associated with a higher tumor stage, metastasis, and recurrence [86,98,99,100,101]. *PTEN* loss leads to the increased activity of the PI3K/AKT/mTOR pathway, which promotes cell survival and proliferation [88,102]. *TP53* loss has also been seen upon disease progression, with mutations observed in approximately 6% to 8% of patients with primary PCa and >28% of patients with metastatic PCa [104,105]. *TP53* is a tumor suppressor gene that plays a critical role in maintaining genomic stability, and its loss or inactivation can lead to the accumulation of additional genetic alterations and increased tumor aggressiveness [103,106,107,108,109]. Another defect associated with PCa aggressiveness and poor prognosis is the loss of the tumor suppressor retinoblastoma (*RB1*) gene, which regulates the cell cycle by inhibiting *E2F* transcription factor activity [89,90,91,92]. Despite the challenges in targeting *RB1* loss in PCa, there is growing interest in developing therapeutic strategies that specifically target this pathway.

Although PCa has been considered an *AR*-driven disease, *AR* gene alterations are very rare in primary PCa, and emerge only after androgen deprivation as a mechanism of castration resistance. In that setting, *AR* amplification has been reported in up to 30% to 40% of cases, and *AR* gene mutations have been identified in approximately 10% to 15% of cases [69]. *AR* splice variants are also frequently observed in CRPC, especially after treatment with second-generation antiandrogens. The most common *AR* variant associated with enzalutamide (ENZA) and abiraterone resistance is *AR-V7*, an *AR* isoform that lacks the ligand-binding domain [110].

Recent studies have shown that combined defects in the tumor suppressors *TP53*, *RB1*, and *PTEN* have been linked to aggressive PCa phenotypes and are potential drivers of lineage plasticity in PCa [26,27,28]. An integrative analysis showed that the null expression of *TP53* through a genomic copy loss or biallelic mutation is seen in ~40% to 50% of metastatic PCa specimens, while biallelic *RB1* inactivation, primarily due to a genomic copy loss, occurs in ~12% of them. Only ~4% of metastatic PCa have been reported to have combined defects at both *TP53* and *RB1* [93,94]. However, combined defects in these genes are more frequent in AVPC patients. Indeed, defects in at least two out of these three genes are included in the NCCN criteria for identifying AVPC patients [95], and can be used to predict benefits from adding carboplatin to cabazitaxel [11], marking them as key lineage-associated markers that are used in clinical practice for decision making. In addition, *TP53* mutations, as defined by a diffuse and intense *TP53* expression, and *RB1* loss, as defined by a lack of expression due to immunohistochemistry, have been associated with small-cell carcinoma morphology [96], which is the prototype of AVPC. *RB1* loss is frequently seen in both the adenocarcinoma and small-cell carcinoma components of mixed tumors [138]. Using PCa mouse models with *PTEN* mutations followed by *RB1* loss, Ku et al. [26] showed that *PTEN* and *RB1* serve as lineage plasticity markers that enhance tumor metastasis, while the additional loss of tumor suppressor *TP53* allows tumors to resist antiandrogen therapy. Using in vitro and in vivo models of human PCa, Mu et al. [27] showed that the loss of function of tumor suppressors *TP53* and *RB1* is mediated by increased *SOX2* levels. *SOX2* is a reprogramming transcription factor, one of the four Yamanaka factors that play crucial roles in differentiation processes [47,97]. The inhibition of *SOX2* expression led to the restoration of *TP53* and *RB1* function, resulting in the increased expression of basal (CK5, CK14, and TP63) and neuroendocrine (SYP, CHGA, and NSE) lineage markers.

A recent examination of a vast repository of PCa patient-derived xenografts (PDXs) [139] that reflects the spectrum of lethal PCa used various high-throughput techniques, including whole-genome sequencing, targeted sequencing, and RNA sequencing (RNA-seq), to gain a better understanding of potential genomic alterations that may contribute to the development of PCa. The heterozygous deletion or amplification of specific genes did not seem to impact gene expression, while most homozygous deletions resulted in null expression. Several known fusions (*TMPRSS2-ERG*, *TMPRSS2-ETV4*, and *SLC45A3-ELK4*) were observed. The combined defects in tumor suppressors *RB1*, *TP53*, and *PTEN* seemed to be the only key players for PCa aggressiveness that could be linked to lineage plasticity events.

Biomarkers that can be used to guide therapy selection are sparse in PCa. *AR-V7* has been proposed as such due to its association with abiraterone and ENZA resistance [112]. However, its use is limited by the rarity of its expression in the pre-abiraterone/ENZA era and its induced expression after exposure to one of these agents. Mutations in DNA homologous recombination repair genes *BRCA2* (the most common), *BRCA1*, *ATM*, and *CHEK2* are early genetic events associated with an increased risk of developing aggressive PCa [73,74,75,87], and are predictive of the response to *PARP* inhibitors [86]. Mutations in DNA mismatch repair (MMR) genes are less frequent, usually seen in the sporadic setting [76,77,78], and are predictive of the response to the immune checkpoint inhibitor pembrolizumab [79].

The genomic alterations observed in PCa are diverse and complex, with many alterations linked to androgen signaling and DNA damage repair pathways. Understanding these genomic alterations is critical for the development of novel diagnostic and therapeutic strategies for PCa. Indeed, the predictive molecular biomarkers currently in use for therapy selection in PCa represent genomic alterations, i.e., homologous recombination gene variants, microsatellite instability, and tumor suppressor gene defects, the latter two frequently identified through immunohistochemistry, an easy and low-cost, widely-available technique. However, genetic alterations alone have failed to fully describe the heterogeneity and complexity of PCa progression, and additional predictive markers for therapy selection are needed to improve patient outcomes. Thus, we and others have hypothesized that a complementary epigenetic network drives lethal PCa progression [140,141,142].

## 3. Epigenetic Changes in PCa Evolution

Whereas genomic alterations can modify gene expression and functioning through changes in DNA sequencing, epigenetic alterations modulate gene expression without any changes in DNA sequencing. Similar to genomic alterations, epigenetic changes can be inherited by daughter cells following cell division. However, in contrast to genomic alterations, epigenetic alterations can be reversed (i.e., due to pharmacologic inhibition or as a response to environmental stimuli) and are, thus, bidirectional, inheritable regulators of gene expression [39]. Epigenetic changes include: (1) DNA methylation, (2) chromatin remodeling through histone post-translational modifications (PTMs) (methylation, acetylation, phosphorylation, etc.), and (3) the effects of noncoding RNAs (ncRNAs, primarily microRNAs (miRNAs), and long noncoding RNAs (lncRNAs)) [143,144]. Epigenetic reprogramming has emerged as an important contributor to cellular processes that drive cancer initiation, progression, and therapy response [145]; thus, epigenetic discoveries can contribute to a better understanding of the underlying events that lead to lineage plasticity and PCa lethality. Table 1 summarizes the most frequently observed epigenetic events in prostate cancer.

### 3.1. DNA Methylation

DNA methylation, the best-studied epigenetic mechanism, refers to the addition of methyl groups to the cytosine residues of CpG dinucleotides [146]. DNA methylation is a dynamic procedure that takes place through enzymes called DNA methyltransferases (DNMTs). In general, CpG dinucleotides are methylated in CpG islands (CpG-dense regions above a threshold of an observed versus expected frequency) in noncoding areas and in the promoters of silenced genes, whereas promoters of expressed genes are unmethylated [147,148]. Here, we describe the most studied DNA methylation changes observed in PCa and highlight those that could potentially be linked to lineage-plastic disease.

Based on the vast majority of altered DNA methylation patterns in PCa, the growth suppressor genes *APC* and *RARβ* and cell adhesion genes *CDH1* and *CD44* are among the most frequently hypermethylated genes in PCa [116,117,118,119,120,121,123,124,125]. The detection of the hypermethylation of the promoter region of the DNA repair gene *GSTP1* (36–100% sensitivity) and cell-cycle-associated gene *RASSF1a* (53–96% sensitivity) [115,128] in biopsies and body fluids (serum, plasma, urine, and ejaculates) has been suggested as a sensitive and specific marker for detecting PCa. Moreover, the hypermethylation of a three-gene classifier (*GAS6/GSTP1/HAPLN3*) has also been proposed as a biomarker for the obtainment of a more accurate PCa diagnosis [149]. Another gene frequently hypermethylated in PCa is *PITX2*, which is a transcription factor involved in several cellular processes, including cell proliferation and differentiation. The aberrant DNA methylation of this gene has been associated with a higher risk of recurrence and metastasis in PCa [150,151]. The writers of DNA methylation (DNA methyltransferases—DNMTs) also represent a relevant object of investigation in PCa. A gradient of DNMT expression levels from low- to high-grade PCa has been reported by our group [152]. In addition, global hypermethylation has been linked to metastatic PCa, and hypomethylation at pericentromeric regions and repetitive sequences has been observed in the same patients [153]. The latter refers to the hypomethylation of certain repetitive sequences, such as long interspersed nuclear element-1 (LINE-1) and satellite 2 (Sat2), both of which have been linked to genomic instability and poor prognosis in PCa [129,130]. However, none of these have been translated into clinical practice, nor have any of them shown predictive significance.

Recently, Loyfer et al. [154] released a DNA methylation atlas of 39 normal cell types. For each cell type, 25 markers were highlighted as being uniquely unmethylated compared to the other cell types. These markers could potentially be used as biomarkers for cell type identification in liquid biopsies and, combined with PCa-specific hypermethylated markers, as presented earlier, could result in better noninvasive tests for tumor detection and classification. In addition, the investigation of the methylation status of these markers in the whole spectrum of PCa (primary, metastatic, and AVPC) could shed light on those that could be used as lineage-plasticity-specific markers that drive the progression of the disease. In 2016, Beltran et al. [9] performed a differential methylation analysis between neuroendocrine (CRPC-NE) and adenocarcinoma (CRPC-Adeno) CRPC subtypes to elucidate the potential epigenetic drivers of PCa evolution. They highlighted four genes (*CCND1*, *GATA2*, *MAPKAPK3*, and *SPDEF*) that were observed to be both hypermethylated and downregulated in the CRPC-NE cohort compared to CRPC-Adeno. Interestingly, Loyfer et al. listed *SPDEF* in the top 1000 markers that seemed to be significantly unmethylated in normal prostate tissue. It is also known that *SPDEF* regulates cell differentiation and has been associated with tumor metastasis in PCa [155]. In addition, Beltran et al. observed eight genes (*ASXL3*, *CAND2*, *ETV5*, *GPX2*, *JAKMIP2*, *KIAA0408*, *SOGA3*, and *TRIM9*) that were hypomethylated and overexpressed in the CRPC-NE cohort compared to CRPC-Adeno [9]. These findings could potentially be linked to lineage plasticity in PCa and potential epigenetic markers.

The DNA methyltransferase inhibitor agent 5-azacytidine has been shown to reverse the global hypermethylation patterns that are observed during cancer development and evolution. This drug has been FDA approved (May 2022) for therapy in newly diagnosed juvenile myelomonocytic leukemia (NCT02447666) [156,157], and >200 clinical trials are currently recruiting patients to test 5-azacytidine alone or in combination for various cancer types, including PCa (https://clinicaltrials.gov/, accessed on 31 May 2023).

### 3.2. Histone PTMs

Chromatin remodeling through histone PTMs represents another epigenetic mechanism frequently altered in PCa. Chromatin can be packed as an accessible euchromatin, which enables gene expression, or as a heterochromatin, which induces gene suppression [158]. This chromatic structure is mainly mediated by histone PTMs, which include methylation, acetylation, ubiquitylation, SUMOylation, and phosphorylation on specific residues of the N-terminal tails of histones [159,160]. In contrast to DNA methylation, which is associated with gene silencing, histone modifications are linked to either gene activation or repression, depending on which residues are modified and the type of modifications present [160,161]. For instance, H3K27ac (the acetylation of lysine 27 on histone H3) and H3K4me3 (the trimethylation of lysine 4 on histone H3) are present at the promoters of transcriptionally active genes, whereas H3K27me3 (the trimethylation of lysine 27 on histone H3) is enriched at repressed gene promoters. Histone methyltransferases (HMTs) and histone demethylases (HDMs) are responsible for adding and removing methyl groups, respectively, and histone acetyltransferases (HATs) and histone deacetylases (HDACs) mediate the addition and removal of acetyl groups to/from histones, respectively [162,163,164]. For example, PRC1 and PRC2 polycomb complexes mediate the trimethylation of histone H3 at lysine 27 residues (marker H3K27me3), resulting in gene silencing and chromatin condensation [165].

The deregulation of histone PTMs modulates gene expression and plays a crucial role in chromatin remodeling. Enzymes that add and remove histone PTMs have been reported to be of clinical relevance in PCa, including the enhancer of zeste homolog 2 (*EZH2*), which catalyzes the addition of methyl groups to histone H3 at lysine 27 (*H3K27*); lysine-specific demethylase 1A (*KDM1A*, also known as *LSD1*), which catalyzes the demethylation of mono- and dimethylated lysines, specifically histone H3 at lysines 4 and 9 (*H3K4* and *H3K9*); and lysine-specific demethylase 7B (*KDM7B*, also known as *PHF8*), which is selective for mono- and dimethylated states [137,166,167]. *EZH2* has been shown to be overexpressed upon PCa progression [44] and >50 clinical trials using EZH2 inhibitors are ongoing (https://clinicaltrials.gov, accessed on 31 May 2023). *KDM1A* and *KDM7B* are also highly expressed in patients with lethal CRPC [136], and numerous clinical trials using *KDM1A* inhibitors are in progress, though none have been reported for *KDM7B*. HDACs are often overexpressed in PCa as well, but while HDAC inhibitors seem to have promising results in hematological malignancies, phase II clinical trials of HDAC inhibitors (vorinostat, pracinostat, panobinostat, and romidepsin) in PCa have failed due to toxicity or disease progression [168]. For the aforementioned histone modifications, EZH2 overexpression has been found to lead to AR silencing in AR-indifferent PCa, transdifferentiation from adenocarcinoma to NEPC, and the activation of lineage-plasticity-related factors [169,170]. The clinical trials of epigenetic modulators that have been or are being tested in PCa can be found in Supplementary Table S1.

### 3.3. Chromatin Remodeling through ncRNAs

Noncoding RNAs (ncRNAs) are RNA transcripts not translated into proteins and can be divided into two groups according to size: miRNAs, which comprise transcripts 18- to 200-nucleotides long, and lncRNAs, which comprise transcripts longer than 200 nucleotides. Gene regulation through ncRNA relies on the binding of ncRNAs to the 3′ UTRs of their target mRNAs, resulting in the RNA degradation or inhibition of translation [171,172]. Aberrant ncRNA expression has been documented in various types of cancer, including PCa [173]. Mapping the ncRNAs of the human genome, as well as their targets, is an ongoing and rapidly expanding effort.

Gene regulation through ncRNA is a promising discovery that could lead to a new biomarker/therapeutic approach. Abnormal miRNA and lncRNA expression has been well documented in most cancer types [174]. Several studies have shown the importance of lncRNAs as modulators of key cellular processes in cancer, and it is believed that many of these transcripts could serve as potential cancer biomarkers [175]. Recent studies indicate that the lncRNAs HOX transcript antisense RNA (HOTAIR), growth arrest-specific 5 (GAS5), PCa gene expression marker 1 (PCGEM1), PCa ncRNA-1 (PRNCR1), PCa antigen 3 (PCA3), and PCa gene expression marker 1 (PCGEM1) interact with *AR* signals for CRPC progression [173,176,177,178,179]. Another extensively studied lncRNA in cancer is PCAT-1, which has been shown to be upregulated in PCa and to promote cancer cell proliferation, migration, and invasion [180,181,182,183]. Moreover, PCAT-1 has been reported to be associated with poor prognosis in PCa patients and could be potentially linked to lineage plasticity [184,185]. Additionally, in a recent publication, Singh et al. [186] highlighted the importance of lncRNA H19 and its association with NEPC, suggesting that upregulated H19 levels can be used as a candidate diagnostic and predictive marker of NEPC and a putative marker of biochemical recurrence and metastatic disease in patients receiving ADT.

In addition to lncRNAs, miRNAs also play a critical role in PCa [187,188,189]. The miRNA miR-21 has been shown to be upregulated in PCa and to promote cancer cell proliferation and invasion by targeting the tumor suppressor *PTEN* [190,191,192]. In addition, miR-34a has been reported to be downregulated in PCa and to inhibit cancer cell proliferation and migration, cooperating with *TP53* [193,194]. Recently, Zhao et al. [195] proposed a panel of five miRNAs (miR-30c-5p/31-5p/141-3p/148a-3p/miR-221-3p) as an independent prognostic biomarker to predict biochemical recurrence after radical prostatectomy.

Besides lncRNAs and miRNAs, other classes of ncRNAs that have been implicated in PCa include circular RNAs (circRNAs), small nucleolar RNAs (snoRNAs), and PIWI-interacting RNAs (piRNAs). The circRNA circHIPK3 has been reported to be upregulated in PCa and to promote cancer cell proliferation and invasion [196,197,198,199]. Moreover, the circRNA circSMARCA5 has been shown to be downregulated in PCa, and its function correlates with the suppression of PCa metastasis [200]. The snoRNA SNORA42 has been reported to be downregulated in PCa and to inhibit cancer cell proliferation and migration [201]. It was also found that piRNA piR-31470 plays a crucial role in the hypermethylation of the promoter of *GSTP1* in PCa [202], while piR-001773 and piR-017184 promote PCa progression by downregulating *PCDH9* expression [203]. While these findings are promising, longitudinal studies across the spectrum of PCa progression are necessary in order to identify biomarkers with high sensitivity and specificity.

**Figure 1:**
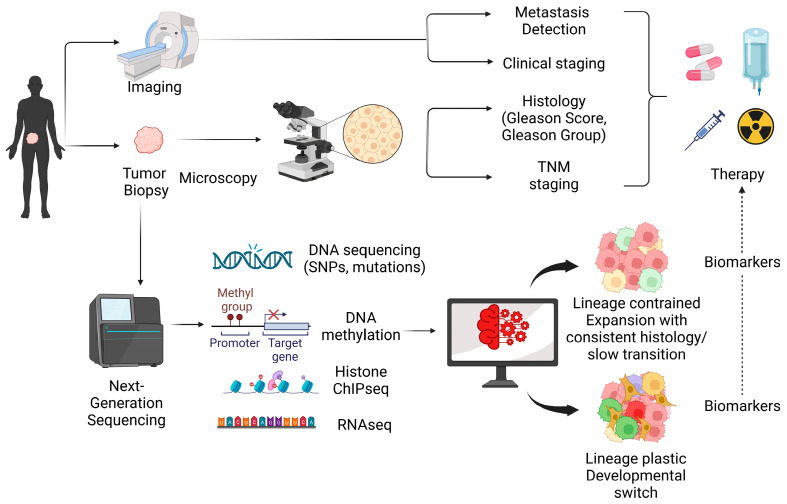
Pipeline of current and future therapy selection approaches in prostate cancer.

It is clear that DNA methylation, histone PTMs, and ncRNAs are important regulators of gene expression in PCa, and that their dysregulation aids in tumor development and progression. Some of the aforementioned markers could potentially be associated with lineage plasticity and serve as candidate epigenetic markers for lineage-plastic PCa. Even though various studies have identified one or a combination of epigenetic players or events as having a prognostic role, none of them are currently used in routine practice. This may be attributed to the complexity of epigenetic regulation and the notion that a single or even multiple epigenetic markers would not be able to fully describe this complexity. Targeting these modifications may represent a promising therapeutic strategy (Figure 1). The lack of the success of epigenetic modulators in solid tumors in general, and PCa in particular, may be attributed to two (not mutually exclusive) factors: (a) the lack of predictive biomarkers for patient selection (which would likely include a network of markers rather than just one or a few) or (b) the need for combination therapy to effectively alter the epigenome. Hence, there is an urgent need to identify epigenetic networks that could serve as both candidate biomarkers and potential therapeutic targets for lineage-plastic PCa.

## 4. PCa Heterogeneity as Defined by Transcriptomic Profiles

Transcriptomics has become a cornerstone in unraveling the intricate heterogeneity of prostate cancer. At a low scale, specific RNA expression profiles can be analyzed with various commercially available platforms to prognosticate specific sets of patients and aid clinical decision making [204]. However, through scrutinizing thousands of genes simultaneously, transcriptomics provides a comprehensive snapshot of gene expression patterns that underlie the diverse characteristics of cancer cells within the prostate tumor microenvironment [205,206,207,208,209]. This may hold promise for even better patient risk stratification in the future.

For example, using a large-scale transcriptomic dataset of 19,470 patients, Spratt et al. were able to identify a low AR-active subgroup in treatment-naïve primary PCa that exhibited molecular characteristics similar to mCRPC [208]. Han et al. were able to identify two luminal (luminal A and luminal S) and two aggressive (AVPC-I and AVPC-M) subtypes, as well as a subtype with mixed transcriptional profiles, with the aggressive subtypes (AVPC-I and AVPC-M) more likely to show docetaxel resistance [210]. Sutera and collaborators performed RNA expression profiling of the primary tumors of patients with mCRPC (stratified as synchronous versus metachronous metastatic disease) [209] and showed that patients who progressed slower had a more hormone-dependent transcriptional profile compared to those with synchronous metastases. These findings strengthen the idea that patients who are destined to follow a more aggressive disease show unique transcriptional profiles, and that the identification of these profiles could inform the clinicians for therapy selection (i.e., earlier use of chemotherapy).

These findings enable a new approach for patient stratification based on transcriptomic subtypes. This stratification offers a more refined framework for personalized treatment strategies, allowing clinicians to tailor interventions according to the specific molecular characteristics of each patient’s tumor. As transcriptomic technologies continue to advance, the exploration of prostate cancer transcriptomic subtypes promises to provide deeper insights into the complexity of the disease, ultimately, paving the way for more effective therapeutic interventions and improved patient outcomes.

However, there are some caveats in the use of transcriptomic analysis in routine practice. Salami et al. modified commercially available molecular scores (cell cycle progression score [211], genomic classifier score [212], and genomic prostate score [213]) by including molecular characteristics of the cellular organization (*FLNC, GSN, TPM2, and GSTM2*), stroma component (*BGN, COL1A1, and SFRP4*) and others, and showed that scores differed between the different grade groups from different tumor foci from the same patient, highlighting PCa tumor heterogeneity at a transcriptomic level [205]. Similarly, Wei et al. performed both genomics and transcriptomics and showed that significant genetic diversity was observed both within different tumor foci from the same patient as well as within different cores from the same tumor focus, underscoring both the intertumoral and intratumoral heterogeneity at the genomic and transcriptomic level for any single patient [206]. These findings have significant implications for using genomic classifiers in precision medicine, especially in the biopsy setting, as a single core from the prostate may not accurately predict the patient prognosis or therapy response. Instead, the range of genomic alterations from multiple cores from the index focus, which is the focus with the most aggressive characteristics, as well as from additional potentially aggressive lesions may be more informative for each patient.

In addition to risk stratification, spatial transcriptomics allows researchers to identify unique gene signatures associated with distinct cellular subpopulations. It also enables the transcriptomic subtyping of the tumor subpopulations, shedding light on the underlying molecular diversity that influences disease progression and therapeutic responses [207,208,210]. Thus, spatial transcriptomics have provided a new approach for unraveling the intricate molecular landscape within the context of tissue architecture, thus, providing a spatially resolved understanding of how genes are expressed across different regions of the tumor microenvironment [214,215,216]. In prostate cancer, spatial transcriptomics offers the opportunity to uncover the heterogeneous distribution of cellular populations, including cancer cells, stromal cells, immune cells, and more. By preserving the spatial context, this method enables the identification of distinct molecular signatures associated with various tumor regions, unveiling potential interplays between different cell types. A recent study showed that a spatial transcriptomic approach enabled the identification of the gene expression heterogeneity observed in a PCa specimen with de novo neuroendocrine PCa and coexisting adenocarcinoma [217]. In addition, using spatially resolved metabolic network modeling, Wang and collaborators analyzed the complexity of the metabolic microenvironment of PCa and showed that malignant-cell-specific metabolic vulnerabilities may serve as candidate targets [218]. In 2018, Berglund et al. [219] used spatial transcriptomics, aiming to map the prostate cancer microenvironment and adjacent areas at a transcriptomic level, and revealed that cancer gene expression could be seen beyond the histologic boundaries of the tumor and that changes in the microenvironment may precede cancer-related genetic changes. These findings have important implications, as abnormal transcriptomics from histopathologically normal areas may alert clinicians to an adjacent or future tumor formation.

Almost 50 years ago, Cunha and Lung, in their stromal–epithelial recombination experiments, showed that prostate epithelial development is dependent on stromal AR signaling [220]. Now, it is well established that stromal–epithelial interactions maintain the homeostasis of prostate tissue, with stromal AR signaling mediating epithelial growth and differentiation and epithelial AR signaling mediating luminal cell function [221]. The stromal microenvironment is altered during prostate cancer development. Cancer-associated fibroblasts (CAFs) have been seen in the tumor stroma, and a microenvironment that enables disease progression, therapy resistance, and metastasis emerged [222,223,224]. Stromal–epithelial crosstalk in prostate cancer has been under investigation for the last several years in order to better understand its role in disease progression and metastasis. Altered stroma exhibits unique molecular profiles that have also been associated with metastasis [225,226]. A low AR expression in stromal cells has been linked to disease progression and/or worse outcome (biochemical relapse, ADT resistance, etc.) [227,228], indicating a protective role of stromal AR. A recent review highlighted the changes observed in AR signaling in tumor stroma that could influence the tumor’s behavior [229]. A transcriptomic analysis has identified changes in the gene expression profile of the stroma adjacent to tumors, with prognostic implications for the patient, indicating that the molecular profile of tumor-adjacent stroma could reveal valuable information regarding PCa diagnosis, progression, and evolution [219,230,231].

In the context of prostate cancer, transcriptomics has been instrumental in revealing the intricate interplay between cancer cells, stromal cells, and immune cells, shedding light on their contributions to disease progression and treatment resistance. As transcriptomic techniques evolve, including single-cell RNA sequencing and spatial transcriptomics, the implementation of these advanced methodologies in studying prostate cancer heterogeneity promises to uncover deeper insights into the molecular dynamics within tumors, enabling more targeted therapeutic strategies and, ultimately, advancing precision oncology approaches.

## 5. Computational and Molecular Perspectives on Lineage Plasticity

Lineage plasticity has long been recognized as a key feature of organ development and tissue regeneration. In recent years, advances in single-cell genomics and transcriptomics have been used to expand our understanding of the mechanisms that underlie lineage plasticity. Most studies have used lineage tracing methods, which label specific cell populations and track their fate over time using in vitro culture systems, genetic manipulation, and transplantation assays [232,233,234,235,236,237,238,239]. While lineage tracing measures allow us to trace longitudinal lineage changes, measures that predict the ability of a cell to switch its differentiation program, undergo dedifferentiation, revert to a more stem-like state, or transdifferentiate into a different cell type remain to be developed. Recent studies have introduced NGS technologies and multiomics as the most promising tools to provide such measures [240,241,242,243,244,245]. Here, we discuss next-generation methods that can be used to develop candidate measures to predict whether a tumor sample shows lineage plasticity features.

Epimutation clocks are hereditary epigenetic alterations that establish fluctuating changes during the progression and evolution of cancer and have been studied in various cancer types. Gabbutt et al. introduced markers that can be used as a fluctuating DNA methylation clock [246] that enables “flip-flopping” between methylated and unmethylated states in colorectal cancer. They further applied this approach to whole blood samples to detect fluctuating DNA methylation clocks and distinguish between acute and chronic leukemias [246], supporting the idea that fluctuating methylation clocks can provide a powerful tool to quantify somatic cell evolution in human tissues. The investigation of epimutation clocks in PCa could give rise to potential markers that could be linked to aggressive disease and lineage plasticity events.

Recent studies in lung adenocarcinoma (LUAD) have shown that multiomics comprising single-cell transcriptomics combined with single-cell epigenomics can reveal distinct and well-described cell states during cancer development and evolution [247,248]. Marjanovic et al. revealed the emergence of a “high-plasticity cell state” (HPCS) with a distinct transcriptional and chromatin profile during the development of LUAD. They analyzed single-cell transcriptomes across the spectrum of LUAD development (seven stages from preneoplastic hyperplasia to LUAD) using genetically engineered mouse models (GEMMs) and showed that a cluster of cells with a highly mixed AT1/AT2 lineage signature was prevalent from early adenomas to fully formed LUAD. These HPCS cells had the most profuse and strong connections to give rise to other cell states and substantial trajectories, and they indeed gave rise to numerous cell states and substantial trajectories when cultured in 3D tumor spheres. In addition, the HPCS expression signature differed from the molecular signature of cancer and normal stem cells [247]. Additionally, a cluster-based pan-cancer analysis across a TCGA collection suggested that the HPCS signature may define more aggressive cancer types associated with drug resistance [247]. Chan et al. [50] analyzed the emergence of HPCS using PCa GEMMs that recapitulated the transition from adenocarcinoma to NEPC with prostate-specific deletion of *TP53*, *RB1*, and *PTEN,* and identified a lineage plasticity signature of mixed luminal–basal gene markers that were unique in highly plastic cells. They demonstrated that the combined defects in these tumor suppressors led to lineage-plastic cell states with a unique mixed luminal–basal molecular signature. In addition, they highlighted the emergence of JAK–STAT and FGFR pathway activation among the programs associated with lineage plasticity and showed that the inhibition of *JAK* and *FGFR* at highly plastic organoids resulted in normal acinar morphology. The authors also compared their findings to human disease using the scRNA-seq of patient samples with CRPC and organoids derived from human CRPC cells, confirming the relevance of their results [50]. Therefore, they showed that the increased activity of *JAK* and *FGFR* was associated with lineage plasticity events. Taken together, these findings strengthen the idea that HPCS represents a lineage plasticity property that is present from the early stages of the disease and can give rise to diverse phenotypic lineages when the tumor’s survival is threatened (i.e., through therapies), leading to poor outcomes. Therefore, measures that can predict the presence of HPCS could be used as candidate lineage plasticity biomarkers and potential therapeutic targets.

Blanco et al. [249] showed that chromatin remodeling represents an epigenetic “memory”, creating inherited chromatin dynamics that give rise to cell states that result in lineage plasticity, which can be described as an inherent cell property rather than as a specific event. Memory cell states are defined by genetic and epigenetic alterations that can be triggered through diverse environmental stimuli, leading to chromatin remodeling and the emergence of the most appropriate cell state at each particular time [250]. In their recent publication, Tang et al. [251] combined an assay for transposase-accessible chromatin with sequencing (ATAC-Seq), chromatin immunoprecipitation sequencing (ChIP-seq), and RNA-seq analyses in PCa cell lines and patient-derived organoids and xenografts to identify four CRPC subtypes with unique chromatin and transcriptional profiles. Those included AR-dependent (CRPC-AR), neuroendocrine (CRPC-NE), Wnt-dependent with low AR expression (CRPC-WNT), and stem-like with low AR expression (CRPC-SCL) subtypes. This study also showed, in agreement with others, that combined defects in the tumor suppressors *RB1*, *TP53*, and *PTEN* were associated with lineage plasticity and aggressive PCa phenotypes [26,27,28,251,252]. In addition, they identified master transcription factors for each CRPC subtype, with *AR* and *FOXA1* being prevalent for CRPC-AR; *NEUROD1* and *ASCL1* for CRPC-NE; *TCF7L12* for CRPC-WNT; and *FOSL1* for CRPC-SCL [251]. A pathologic, genomic, and marker gene expression analysis provided validation of the four subgroups, with CRPC-AR showing high levels of AR expression and score, CRPC-NE having high SYP expression and a NE-morphologic score, CRPC-WNT specimens showing elevated *AXIN2* expression, and the CRPC-SCL subtype being defined by high CD44 expression levels. Formaggio et al. [253] implicated the overexpression of three (*SOX2, OCT4*, and *MYC*) out of the four Yamanaka factors in the dedifferentiation process of lineage plasticity observed in PCa, while the loss of the Yamanaka factor KLF4 was associated with tumor evolution [253].

Thus, early data support the notion that PCa may be classified based on lineage plasticity through the presence of HPCS, as well as epigenetic markers that may characterize these states. The presence of HPCS in a tumor sample may potentially be linked to lineage plasticity and could indicate aggressive disease with poor outcomes. The identification of epigenetic markers and mechanisms that enable lineage plasticity in some, but not all, patients could represent a fundamental milestone for diagnosis, prognosis, and targeted therapy. The development of bioinformatic tools that focus on HPCS identification is likely be critical in this effort.

## 6. Bioinformatic Tools for Lineage Plasticity Signatures and Measures

Bioinformatic tools have played a pivotal role in the identification of biomarkers, as well as the development of molecular signatures in cancer [254,255,256,257,258]. Through the analysis of large datasets, these tools enable the identification of genes and pathways that are dysregulated in cancer cells. The identification of biomarkers through bioinformatic analyses enhances the development of targeted therapies and personalized medicine [259,260,261]. In addition, enrichment analyses provide mechanistic insights into the underground biology of the development and evolution of the disease [262,263]. Overall, the integration of bioinformatics into cancer research has significantly improved our understanding of the molecular mechanisms underlying cancer and has the potential to improve patient outcomes. Here, we focused on bioinformatic tools that could be incorporated into a multiomics approach to identify lineage plasticity measures and signatures. Table 2 provides a list of bioinformatic tools that could potentially be used to generate measures of lineage plasticity.

### 6.1. Genomics

A variety of genomic bioinformatic tools are available to describe mutations, including copy number alterations (CNAs), which refer to changes in the copy number of genomic regions, such as amplifications and deletions, and copy number variations (CNVs), which are more comprehensive and encompass a broader range of structural alterations in the genome, including CNAs as well as duplications and complex rearrangements across bulk and single-cell data. These tools enable the identification of specific genomic drivers of cancer [267,269,271,272,301]. Importantly, an integrated multiomics approach would be needed to associate these genomic drivers with lineage plasticity. Here, we described some of the most well-known tools used for CNV identification in genomic data, highlighting those that could be used for single-cell sequencing analyses. Single-cell DNA sequencing (scDNA-seq) allows for single-cell resolution, but has limitations regarding DNA quantity (approximately 6 pg) that are not applicable for whole-genome sequencing [302]. There are methods to overcome these limitations (i.e., multiple displacement amplification, multiple annealing, and looping-based amplification cycles) when amplification bias arises. To identify reliable CNAs, specifications, including genomic uniformity, depth of coverage, and throughput, are important parameters. A higher depth of coverage enables the detection of smaller CNAs with a higher resolution of CNA boundaries [302]. The throughput of scDNA-seq refers to the number of cells that can be simultaneously sequenced, as well as the time needed to complete the sequencing procedure. A high throughput enables a large number of cells to be sequenced, resulting in a more detailed understanding of those cells.

MuTect [264] is a powerful computational tool in cancer genomics. Developed by the Broad Institute of MIT and Harvard, MuTect is specifically designed for the detection of somatic mutations using tumor–normal paired samples obtained from NGS data. It compares the genetic profiles of tumor and normal samples to identify and differentiate true somatic mutations from sequencing artifacts and germline variants. This process includes four key steps: the removal of low-quality sequence data, variant detection, filtering and the removal of false-positive results, and the identification of somatic versus germline mutations. This tool has been used for the detection of somatic mutations in various cancer types, including PCa [83,139,265,303]. In PCa, MuTect has been used to identify genomic variations in African populations, which showed an elevated tumor mutational burden in African men with treatment-naïve, high-risk PCa [304]. In addition, using the MuTect package, Hong et al. [305] showed that enrichment of *TP53* mutations was linked with metastatic potential in blood samples from patients with metastatic PCa. Therefore, MuTect is a solid tool that can be used for mutational screening and revealing potential mutational drivers of lineage-plastic PCa.

Maftools is an R (Bioconductor) package that provides a comprehensive suite of tools for the analysis and visualization of mutations in cancer genomics data [267]. It provides functions (“plotmafSummary” and “maftoolsSignatur”) to analyze the mutational burden and mutation signatures. Mutation annotations can also include additional information, such as gene annotations, functional impact predictions, and known cancer driver genes. In addition, the package includes advanced visualization functions to generate high-quality plots, such as oncoplots, waterfall plots, and heatmaps, to aid in the identification and interpretation of driver mutations and their associated clinical outcomes. Maftools is a powerful, versatile, and user-friendly tool for the analysis, interpretation, and visualization of somatic mutation data in cancer genomics. By providing a range of visualization and analysis functions, it allows researchers to gain insights into cancer’s genetic mechanisms and to identify potential therapeutic targets. While Maftools was originally designed for bulk DNA-seq analyses, it can potentially be applied to the analysis of somatic mutations in scDNA-seq data, particularly if the data have been aggregated to generate a mutation frequency matrix or mutation annotation format (MAF) file. Maftools has also been used to detect mutations in PCa specimens, as well as for meta-analyses [268,306]. Therefore, it provides an additional tool for identifying mutations that could be linked to lineage-plastic PCa. To achieve this, a selection of the most appropriate cohorts is mandatory to enhance the reliability of the results at a genomic level.

CopyKit is an R package designed to preprocess and analyze single-cell CNV genomic data in advance of the detection and visualization of CNVs, including those that occur at low allele frequencies and in subclonal populations [269]. CopyKit enables the analysis of the copy number substructures of tumor samples, as well as in furthering the investigation into the intratumoral heterogeneity that is frequently seen in PCa [307]. It also provides a quality control module to process high-quality aneuploid cells for downstream analyses. It marks euploid cells and then filters low-quality cells. CopyKit employs a Bayesian framework for CNV detection, which allows for the accurate estimation of the copy number and allele frequency, as well as the assessment of uncertainty and false discovery rates. The package includes a range of visualization tools, such as heatmaps and scatter plots, which enable the exploration and interpretation of CNV data at different scales. CopyKit is a user-friendly tool that can facilitate the analysis and interpretation of CNV data in a range of genomic sequencing applications, including single-cell sequencing and tumor heterogeneity studies. It provides the advantage of detecting CNVs even in low allele frequencies and in subclonal populations, enabling a better characterization of heterogeneous PCa samples.

HMMcopy is a hidden Markov model (HMM)-based package that provides a wide range of tools for the preprocessing, analysis, visualization, and downstream analysis of genomic data [301]. HMMcopy provides a set of functions and algorithms for detecting and quantifying CNVs from sequencing data, particularly in the context of scDNA-seq. The main advantage of HMMcopy is its ability to accurately detect low-frequency CNVs and mosaic events, which can be missed using other methods. In addition, it enables the simultaneous inference of the segmentation and absolute copy number [302]. After reading the sequencing data, segmentation takes place; then, based on the segment data, a HMM is trained to infer the most likely copy number states. While HMMcopy has mainly been used for other types of data (CGH data), it has also been applied to large-scale scDNA-seq data. The main limitation of HMMcopy is the manual calibration of many parameters and its unreliable detection of ploidy, which often results in an inaccurate copy number estimation [270]. HMMcopy is a frequently used tool for studying CNVs in prostate cancer and has been used to reveal potential biomarkers of lethal outcomes in patients with PCa [308,309,310].

CHISEL (Copy number Haplotype Inference in Single cells using Evolutionary Links) is the first tool for allele-specific and haplotype-specific copy number inference in scDNA-seq data [271]. Using a matched normal or pseudonormal sample derived from diploid cells, CHISEL can overcome the low coverage of scDNA-seq data to detect CNAs by amplifying the weak SNP signal. It can also calculate the B-allele frequency (BAF) in genomic regions of approximately 5 Mb by combining a reference-based algorithm with a novel algorithm to phase short haplotype blocks in each cell. CHISEL provides a hierarchical clustering of cells with similar genomic characteristics, as well as tools for gene enrichment and downstream analyses. It can also be integrated with other single-cell sequencing data types such as RNA-seq to better understand tumor evolution and lineage plasticity. CHISEL offers different Python commands to run either the entire pipeline with all the steps or only some specific steps (chisel, chisel_nonormal, etc.).

### 6.2. Transcriptomics

Bioinformatic approaches that aim to analyze scRNA-seq data represent the vast majority of tools for characterizing lineage plasticity, either through tracing trajectories, using machine learning algorithms, or identifying HPCSs. The Waddington optimal transportation (Waddington-OT) model is a well-known algorithm [273] based on the idea that cells are randomly drawn from a probability distribution of gene expressions and that each cell has a distribution of likely origins and possible fates. This framework uses longitudinal scRNA-seq data to understand how these probability distributions change over time. It applies the mathematical approach of optimal transport to investigate the process of cellular reprogramming after a transient overexpression of transcription factors [47] to answer various questions, such as what types of cells arise during reprogramming, which developmental paths lead to reprogramming and alternative fates, and what intrinsic factors and cell–cell interactions play a role in this process. The insights of this framework could potentially improve the efficiency of cell reprogramming toward a desired outcome. Regarding the application of the Waddington-OT model to scRNA-seq data, the development of the code first requires the loading and normalizing of patient data. Then, highly variable genes are selected and the parameters of the model are defined. Next, random initial cell states are generated and the developmental landscape and gradient are identified. Lastly, the “OTclust” function is used to find cell trajectories based on the Waddington-OT model. The Waddington-OT model was used in a recent publication based on the identification of HPCSs in lung carcinoma [247], and can also be used for longitudinal studies in PCa to highlight cell populations with HPCS characteristics. However, it requires a sequential scRNA-seq data collection, which elevates the experimental cost.

Similar to the Waddington-OT model, Forrow et al. [242] developed an algorithm called Lineage-OT that aims to combine lineage tracing and trajectory inference in a unified manner. The framework utilizes mathematical tools from graphical models and optimal transport to reconstruct developmental trajectories from time courses with snapshots of both cell states and lineages. According to the findings, incorporating lineage data into the framework results in improved accuracy in tracking complex state transitions with even fewer measured time points. Furthermore, the integration of lineage tracing with trajectory inference could enable the accurate reconstruction of developmental pathways that are difficult to recover using state-based methods alone. Therefore, optimal transportation models could potentially be used, not only to define lineage tracing in longitudinal samples, but also to develop predictor models of whether a tumor is destined to progress in aggressive phenotypes or whether it is likely to remain with consistent lineage even after its expansion.

Monocle 2 is an R package that focuses on cell fate identification via reversed graph embedding (RGE), a machine learning approach for a more accurate reconstruction of single-cell trajectories [274,275]. The pipeline works on scRNA-seq data and includes: (a) differentially expressed gene identification for each cluster using *t*-distributed stochastic neighbor embedding (t-SNE) dimension reduction followed by density peak clustering [311]; (b) pseudotime trajectory reconstruction using the DDRTree RGE algorithm, which is performed to lead at a “principal graph” [312,313]. The principal graph is shaped as a curve with branches, where the breakpoints are the “intermediate” datasets and the branches are different cellular states/outcomes [274]. In addition, a branch expression analysis modeling (BEAM) algorithm is used to identify genes with significant branch-dependent expressions in order to determine the intermediate datasets [314]. After reading and preprocessing the data, the developmental trajectory is generated using Monocle 2’s DDRTree algorithm. Monocle 3 has now been released and further information on installing and using this version is available at https://cole-trapnell-lab.github.io/monocle3/ (accessed on 1 July 2023). The publication of the updated Monocle version is not yet available, as it is currently in the beta phase of its development. However, this tool could be powerful for the identification of cellular states within the same dataset compared to the Waddington-OT and Lineage-OT models. Monocle 2 shows a significant advantage of lineage tracing within the same sample, enabling the investigation of tumor heterogeneity within a single specimen. This would be compelling for the identification of HPCS populations in PCa specimens that could determine the presence or absence of lineage plasticity events.

The Seurat R package is a popular toolkit for the analysis, visualization, and exploration of scRNA-seq data [276]. This tool could be employed to identify and quantify lineage plasticity signatures by providing a comprehensive toolkit for single-cell data analyses, enabling researchers to uncover cellular heterogeneity and developmental trajectories. It includes many techniques and methods for data transmutation, detection, infiltration of doublet genes (scDblFinder), and data normalization. For a downstream analysis, various Seurat-supported techniques are available, such as the principal components analysis (PCA), clustering, and the UMAP package, which simplifies data visualization by condensing it into two dimensions. Cell cluster identification is achieved through the “FindClusters” function, employing a shared nearest neighbor (SNN) modularity optimization-based clustering algorithm. SNN compares the nearest neighbors of each cell and defines clusters based on the similarity of their local neighborhoods. Thus, the “FindClusters” function can effectively group cells with similar gene expression profiles, which often represent distinct cell types or states. Additionally, the Seurat package offers an accessible and computationally efficient gene signature function called “AddModuleScore” [278,280]. The module score represents the average expression of a group of genes (usually related to a specific biological function or pathway) in each cell, adjusted for the overall gene expression level in that cell. This allows researchers to compare the activity of certain gene sets between different cell types or conditions. AddModuleScore has been extensively used to test molecular signatures in PCa [315,316,317] and could be further used for molecular signatures linked with lineage-plastic disease.

Unlike the “AddModuleScore” function in the Seurat package, which normalizes its scores using the dataset’s average expression, UCell [279] uses the Mann–Whitney U analysis to calculate gene signature scores. This package allows researchers to investigate the activity of specific gene sets in individual cells, facilitating the identification of cellular subpopulations and uncovering biological processes or pathways that are active in distinct cell types or states. Thus, gene signature scoring algorithms are accessible and assist in enhancing signatures as potential biomarkers for various conditions, such as lineage plasticity. A molecular signature indicative of lineage plasticity could significantly improve cancer prognosis and therapeutic markers for PCa.

CytoTRACE [281] is one of the first tools that aims to measure the presence of lineage plasticity and improve our understanding of cellular dynamics in cancer progression. It is a computational method designed to analyze scRNA-seq data to predict the differentiation potential of individual cell clusters. It also leverages single-cell gene expression data to rank cells based on their differentiation states, from undifferentiated stem cells to more differentiated cell types. CytoTRACE can potentially serve as an independent measure of lineage plasticity, as it calculates the number of expressed genes per cell using single-cell transcriptomic data. With its extensive coverage, including over 18,000 annotated gene sets, it allows for the identification of 52 experimentally determined cell states. This comprehensive approach enables researchers to investigate cellular hierarchies and differentiation potentials, making CytoTRACE a valuable tool for assessing lineage plasticity in various biological contexts. Furthermore, using single-cell transcriptomic and bulk ATAC-seq of human paraxial mesoderm lineage phenotypes, Gulati et al. determined that less differentiated cells possess larger gene counts, which also reflects a more open chromatin accessibility profile compared to well-differentiated cell states. This algorithm could potentially be applied to identify HPCS clusters during the evolution of PCa and set a fundamental milestone in the HPCS identification of lineage-plastic PCa.

### 6.3. Epigenetics

Epigenetic studies aim to describe the epigenomic landscape of a tumor to better elucidate the underlying biology of cancer evolution. ChIP-seq of histone markers (i.e., H3K27ac and H3K4me1) or ATAC-seq can reveal the chromatin accessibility of a cell state during tumor progression and evolution, while DNA methylation sequencing (DNAme-seq) can be used to identify hypermethylated and hypomethylated regions that can be correlated with disease state or other properties, including lineage plasticity [281,285,286,287,288,289,290,291,292,318,319,320]. These analyses can reveal candidate epigenetic biomarkers that are linked to lineage plasticity. Here, we discussed the most common packages that are used for ChIP-seq, DNAme-seq, and ATAC-seq analyses.

#### 6.3.1. ChIP-Seq Analysis Tools

The model-based analysis of ChIP-seq (MACS) remains the most well-known tool for identifying enriched regions of transcription factor binding and histone modifications from ChIP-seq data [282,283]. The updated version MACS2 uses a combination of modeling and peak merging strategies to accurately identify enriched regions [284]. MACS2, in addition to Poisson distribution, incorporates a local lambda model to account for local biases in the data and a dynamic threshold to control for false positives. MACS2 can detect both broad and sharp peaks in contrast with MACS, which was designed for sharp peaks. Additionally, MACS2 provides options for conducting a downstream analysis, such as peak annotation and motif discovery. MACS2 has been used in PCa to identify chromatin regions that are altered upon the acquisition of ENZA-resistance and enabled the selection of the appropriate therapy to target ENZA-resistant CRPC [321]. In addition, recent studies have used MACS2 to describe the epigenetic landscape of primary and aggressive subtypes of PCa, enabling the identification of candidate therapeutic targets [322,323]. Further investigations of the epigenetic landscape of lineage-plastic PCa using MACS2 could inform candidate epigenetic markers and therapeutic targets.

SICER (spatial clustering for identification of ChIP-enriched regions) is a widely used peak-calling method for ChIP-seq data [285,324]. SICER uses a clustering approach to identify enriched regions based on spatial proximity and divides the genome into nonoverlapping windows of size *w* and identifies regions of enrichment. Then, it applies a clustering algorithm to group these regions into larger enriched domains. To complete this operation, an algorithm is developed in Python using the parameters of the window size and a threshold approach allows SICER to identify smaller, closely spaced enriched regions that lead to high sensitivity and specificity. SICER also incorporates an FDR estimation method to control for multiple testing, providing a measure of statistical significance for the identified regions. Additionally, SICER can detect both broad and sharp peaks, whereas MACS is optimized for sharp peaks. Using SICER, Coleman et al. [325] determined the epigenetic landscape of *BRD4* binding sites and identified BET bromodomain inhibitor sensitivity through *MYC* suppression, while Dhar et al. [326] introduced the MTA1/Epi-miR-22/E-cadherin axis as an important metastasis-promoting epigenetic signaling pathway in PCa.

ChIPseeker is an R Bioconductor package that is also well known for the annotation and visualization of ChIP-seq data [286]. ChIPseeker annotates ChIP-seq peaks to genomic features, including genes, exons, introns, promoters, and enhancers. It also provides custom annotation functions that can be used to annotate user-defined genomic features (e.g., “annotatePeak”). Its visualization functions include heatmaps (“plotHeatmap”), profiles, and genome browser tracks, allowing users to explore and visualize ChIP-seq data in a variety of ways. ChIPseeker also provides the opportunity for the downstream and enrichment analysis of the genes annotated by the ChIP-seq peaks, which could provide insight into the biological processes and pathways regulated by the transcription factors or histone modifications of interest. The main difference between MACS and ChIPseeker is that MACS focuses on peak calling and the identification of enriched regions of ChIP-seq data, while ChIPseeker is primarily used for the annotation and visualization of ChIP-seq peaks. Recent studies used ChIPseeker to screen chromatin alterations upon drug administration in PCa samples [169,327].

#### 6.3.2. DNAme-Seq Analysis Tools

Bismark is a widely known tool for the alignment and analysis of DNAme data, providing simultaneous read mapping and methylation calling in a single command [287]. It has been designed in bash language to perform the alignment of bisulfite-treated reads to a reference genome provided by the user and to available public databases. It then discriminates the methylation status between cytosine residues in CpG, CHG, and CHH contexts, enabling the visualization of methylation data to interpret the results. Bismark is a highly configurable tool that allows users to adjust its parameters, such as alignment stringency, quality filtering, and read trimming. It also provides options for filtering out PCR duplicates and calculating differential methylation between samples. Bismark is available as a command-line tool and can be run on Linux, MacOS, and Windows operating systems. It requires a Perl [328,329] programming language, as well as the installation of either the Bowtie [330,331] or Bowtie 2 [332,333] alignment programs. Bismark can work with whole-genome bisulfite sequencing (WGBS) data and representation bisulfite sequencing (RRBS) data. It has been extensively used to study the epigenetic landscape of the whole spectrum of PCa providing important insights into hypomethylated and hypermethylated genes in each cell state [334,335,336,337].

BS Seeker is another tool for the alignment of bisulfite-treated reads to a reference genome. It can also identify methylated and unmethylated cytosines at a single-base resolution [288]. To perform these functions, lists containing the command and its arguments to run BS Seeker for alignment or methylation calling are necessary. BS Seeker also uses Bowtie to map the bisulfite reads generated from WGBS or RRBS data. It includes the post-procedure removal of low-quality mappings based on the number of mismatches. While Bismark uses a bisulfite-aware alignment algorithm that accounts for the effects of bisulfite treatment on the DNA sequence, BS Seeker uses a two-step alignment approach that first aligns the reads to an unconverted reference genome and then uses a bisulfite conversion algorithm to generate a converted genome for the alignment of the bisulfite-treated reads. This approach may provide greater flexibility in the choice of a reference genome and alignment algorithm, but may also introduce some biases or inaccuracies in the conversion process.

MethylKit is an R package that has been designed for the analysis and visualization of DNA methylation data [289]. It provides a variety of tools for the identification of differentially methylated regions (DMRs) from bisulfite sequencing data, as well as functions for the downstream analysis of DMRs. It includes algorithms for data normalization and visualization tools, such as heatmaps, scatter plots, and density plots, for the interpretation of findings. In addition, MethylKit enables the annotation of the genomic and gene ontology analyses of the DMRs. In contrast to Bismark and BS Seeker, which focus on alignment, MethylKit focuses on the analysis and visualization of the results. MethylKit has been used in PCa for the identification and annotation of differentially methylated sites that could reveal potential epigenetic markers linked to different disease states [9,335,338].

#### 6.3.3. ATAC-Seq Analysis Tools

Many tools that can be used for ChIP-seq analyses are also applicable for bulk ATAC-seq analyses, including MACS2 and ChIPSeeker [284,286]. Here, we highlighted a number of tools that could be used to analyze single-cell ATAC-seq data.

Single-Nucleus Analysis Pipeline for ATAC-seq (snapATAC) is one of the few packages designed for conducting a comprehensive single-cell ATAC-seq (scATAC-seq) analysis [291]. SnapATAC provides a variety of tools, including the alignment of the read to a reference genome, quality control, peak calling, visualization, and clustering. It allows for the identification of cellular heterogeneity by comparing chromatin accessibility profiles between cells. In addition, the tool supports the integration of single-cell gene expression data, which allows for the identification of cell types and states based on chromatin accessibility and gene expression patterns. It can also predict enhancer–promoter interactions and enables batch correction, differential accessibility analysis, identification of lineage trajectories and key transcription factors. In PCa, snapATAC has been used to study chromatin sites that are shared in low-grade PCa and lost in high-grade samples [339].

Cellcano is a recently developed tool for the inference of cellular hierarchies in scATAC-seq data [292]. It uses a two-round supervised learning algorithm to identify cell types. First, it uses the reference dataset to train a multilayer perceptron to identify anchor cells in the target dataset. Then, using these anchor cells, it trains a knowledge distiller model (KD model) [340] to learn the relationships between chromatin accessibility profiles and cell types. The trained KD model is then applied to predict the cell types of non anchor cells. Cellcano is a recent tool and no publications on PCa have been published yet. However, it is a promising tool for cell type annotation in scATAC data, and could be used to provide insights into tumor heterogeneity observed at the chromatin level.

Signac is a recent R tool that has been designed for the analysis and visualization of scATAC-seq data [293]. It provides a variety of tools, including peak calling, quality control, visualization, clustering, and integration with scRNA-seq data. It also enables the identification of differentially accessible peaks, enriched motifs, key transcription factors, and gene annotation of the peaks. Importantly, Signac has been designed to interact with the Seurat package, enabling multiomics analyses. In PCa, Signac has been used to identify epigenetic markers of metastatic potential, which is in the same direction as the goal of identifying markers of lineage-plastic disease [341,342].

EpiAnno is a Python tool that has recently been developed for scATAC-seq data analyses using a probabilistic generative model and a Bayesian neural network [294]. The model is designed to embed cells into a latent space where each cell type corresponds to a Gaussian mixture distribution. EpiAnno characterizes cell heterogeneity and has shown accurate results for within-dataset and cross-dataset annotations. The trained EpiAnno and learned cell-embedding parameters are interpretable and can reveal biological insights through a tissue-specific expression enrichment analysis, partitioned heritability analysis, cell type-specific enhancer identification, and cell type-specific cis-coaccessibility analysis. Since EpiAnno is a recently developed tool, no publications in PCa are available. However, it provides important features for studying intratumoral heterogeneity, which is one of the most important characteristics of PCa.

### 6.4. Enrichment Analysis

Gene enrichment analyses provide descriptions of upstream and downstream regulatory pathways and associated molecules, which are necessary to elucidate the biological background of cancer development and evolution. Gene enrichment analyses can be performed using computational software (i.e., GSEA and IPA), while publicly available databases (i.e., NCBI/NIH, GEO, and TCGA) provide an essential repository of well-defined data and molecular signatures that could be used in this setting [295,296,343,344].

The gene set enrichment analysis (GSEA v. 4.3.2) is a powerful global tool for the characterization of cellular functions and pathway enrichment, as well as endogenous and exogenous changes and the relations between the genes of individual datasets. GSEA requires a ranked list of genes through differential expression, as well as the selection of the window of the ranked list and preferred parameters for analysis [345]. In this way, GSEA provides the analysis of an extended list of gene sets with information regarding the expression status (upregulation or downregulation) of the input datasets. The vast majority of publications have used GSEA to perform pathway enrichment analyses, and it has provided important insights into the underlying mechanisms of PCa development and evolution [346,347,348,349,350,351,352,353].

Ingenuity Pathway Analysis (IPA Summer Release (2023)) is another software broadly used for gene set analyses, aiming to elucidate upstream and downstream biological events. In contrast to GSEA, IPA predicts the master regulators for each upstream or downstream regulatory pathway and potential targets for drug development and experiments [296]. While both software are easy to use with detailed tutorials, GSEA has the advantage of being free of fees, while IPA requires a subscription fee. IPA has been extensively used in PCa to study canonical pathways and molecule interactions in different disease states [354,355,356,357,358,359].

Improvements in this field are continuous, and online platforms for enrichment analysis have already emerged. Enrichr analysis is an integrative web-based tool for enrichment analysis that includes one of the biggest lists of gene set libraries [297,298]. Enrichr visualizes the enrichment results as clustergrams and includes information about differentially expressed genes after drug, gene, disease, and pathogen perturbations. Another web-based tool, FLAME [300], allows for the input of multiple gene lists with a parallel exploration and analysis, and utilizes STRING’S API [360] to generate interactive protein–protein interaction (PPI) networks. FLAME provides a visual analytics approach with adjustments and parameter options in addition to heatmaps, bar charts, Manhattan plots, networks, and tables.

## 7. Conclusions and Future Directions

The identification of the determinants of lineage plasticity and the definition of a measurable metric of such signatures at the molecular level in PCa are predicted to translate into prognostic and predictive biomarkers of the disease, as well as new therapeutic strategies, particularly with the goal of addressing chemoresistance. NGS technologies are promising tools for the development of such measures. Single-cell NGS technologies studying GEMMs, human tissues, and PDXs allow us to perform longitudinal lineage tracing across the different cell states that arise during tumor development, therapy resistance, migration, and metastasis [361,362,363]. As presented in this review, NGS assays have been used to provide high-resolution information relevant to intratumoral heterogeneity and the tumor microenvironment at genetic, transcriptional, and epigenetic levels and to identify crucial factors and cell states that promote tumor progression, therapy resistance, and migration [364,365,366,367,368].

Historically, microscopy methods have been tried and tested for their efficacy as a robust tool for analyzing cancer-related challenges. The evolution of NGS, the emergence of big data, and the plethora of machine learning tools create a highly promising molecular avenue for cancer analysis. Once technological and analytical hurdles have been resolved, it is predicted that the application of DNA-seq in molecular pathology evaluation of tumors could eventually rival that of the microscope [369]. Moving forward, the research community should focus on integrating these two aspects to achieve a system-level understanding of lineage plasticity, which would yield more reliable and comprehensive results.

Combined defects in the tumor suppressors *RB1*, *TP53*, and *PTEN* seem to be significant for PCa lineage plasticity events. In addition, epigenetic alterations, including the overexpression of epigenetic modulators such as *EZH2* and *SOX2*, seem to be involved in tumor evolution as components of lineage plasticity. Epimutation clocks similar to those proposed in recent studies [246,370] remain to be characterized in PCa. Furthermore, the presence of HPCS clusters could be used as candidate biomarkers for lineage plasticity, which is linked to aggressive phenotypes. The characterization of lineage-plasticity-associated chromatin remodeling could also represent a fundamental milestone for understanding and targeting lineage-plastic PCa. Unique signatures identified through enrichment analyses and signature scores could inform the characterization of lineage plasticity, revealing additional targets to disrupt the driver events.

## Figures and Tables

**Table 1:** Frequent gene alterations in prostate cancer.

Gene	Type of Alteration	Frequency in PCa	Relevance to PCa	References
*ETS* *TMPRSS2-ERG* *TMPRSS2-ETV1/4* *SLC45A3-ELK4*	Fusion	24–79%	Enhances tumorigenesis and disease progression	[64,65,66,67,68,69,70]
*SPOP* *BRCA1* *BRCA2* *ATM* *CHEK2* MMR genes	Mutation	12% 0.4–0.9% 3–5.3% 1.6% 1.9% <1%	More often in aggressive disease	[71,72,73,74,75,76,77,78,79,80,81,82,83,84,85,86,87]
*PTEN* *RB1*	Loss	15–20% ~1–10%	Associated with aggressive disease	[11,26,27,28,86,88,89,90,91,92,93,94,95,96,97,98,99,100,101,102]
*TP53*	Mutation	6–8% in primary disease >28% in metastatic disease	More frequent in aggressive disease	[26,27,28,95,103,104,105,106,107,108,109]
*AR*	Splice variant Amplification Mutation	varies 30–40% 10–15%	Associated with response and resistance to ARSI therapies	[69,110,111,112]
*GSTP1* *RASSF1A* *APC* *RARβ* *CDH1* *CD44* *PITX2*	Hypermethylation	30–90% 53–83.6% 27–84% 53–96% 27% 32% 1–80%	Downregulation of target genes with a potential higher risk of recurrence and metastasis	[113,114,115,116,117,118,119,120,121,122,123,124,125,126,127,128]
*LINE-1* *SAT2*	Hypomethylation	Not determined	Linked with poor prognosis	[129,130]
*EZH2* *KDM1A* *KDM7B*	Overexpression	Not determined	Affects histone post-translational modifications and associated with poor recurrence-free survival	[64,91,131,132,133,134,135,136,137]

**Table 2:** Bioinformatic tools that could be used for genomic, transcriptomic, and epigenetic enrichment and downstream analysis.

Tool	Description	GitHub Link (If Available)	References
**Genomics**			
MuTect	Detection of somatic mutations using tumor–normal paired samples obtained from NGS data	https://github.com/broadinstitute/mutect (accessed on 1 July 2023)	[264,265,266]
Maftools	Analysis and visualization of mutations in cancer genomics data	https://github.com/PoisonAlien/maftools (accessed on 1 July 2023)	[267,268]
CopyKit	Preprocessing and analysis of single-cell CNVs	https://github.com/navinlabcode/copykit (accessed on 1 July 2022)	[269]
HMMcopy	Inference copy number alterations and single-cell CNV analysis	https://github.com/shahcompbio/hmmcopy_utils (accessed on 1 July 2023)	[270]
CHISEL	Allele-specific and haplotype-specific copy number inference of scDNA-seq data	https://github.com/raphael-group/chisel-data (accessed on 1 July 2023)	[271]
Ginkgo	Analysis of scDNA-seq data as well as post-processing steps, such as downstream analysis and phylogenetic trees	https://www.ginkgobioworks.com/ (accessed on 1 July 2023)	[270,272]
**Transcriptomics**			
Waddington-OT	Cellular fate determination and differentiation	https://github.com/zsteve/gWOT (accessed on 1 July 2023)	[273]
Lineage-OT	Lineage tracing and trajectory inference	https://github.com/aforr/LineageOT (accessed on 1 July 2023)	[242]
Monocle 2	Cell fate identification through single-cell trajectories	https://github.com/cole-trapnell-lab/monocle2-rge-paper (accessed on 1 July 2023)	[274,275]
Seurat R package	Single-cell RNA-seq data analysis, including quality control, preprocessing, exploratory analysis, and downstream analysis	https://github.com/satijalab/seurat (accessed on 1 July 2023)	[276,277]
AddModuleScore function	Biological pathway analysis, gene signatures, or functional modules in individual cells, and for downstream analysis, such as identifying cell states or characterizing cellular heterogeneity based on pathway or module activity.	https://github.com/satijalab/seurat/blob/master/man/AddModuleScore.Rd (accessed on 1 July 2023)	[276,278]
UCell	Gene signature scores	https://github.com/carmonalab/UCell (accessed on 1 July 2023)	[279,280]
CytoTRACE	Quantification of cellular trajectories and differentiation of cell states using (scRNA-seq) data	https://github.com/pinellolab/pyrovelocity/blob/master/pyrovelocity/cytotrace.py (accessed on 1 July 2023) https://cytotrace.stanford.edu (accessed on 1 July 2023)	[281]
**Epigenetics**			
MACS (Model-based Analysis of ChIP-Seq)	Chromatin immunoprecipitation sequencing (ChIP-seq) data analysis	https://macs3-project.github.io/MACS/ (accessed on 1 July 2023)	[282,283,284]
SICER (Spatial Clustering for Identification of ChIP-Enriched Regions)	Peak calling in ChIP-seq data	https://github.com/zanglab/SICER2 (accessed on 1 July 2023)	[285]
ChIPseeker	Annotation and visualization of ChIP-seq data	https://github.com/YuLab-SMU/ChIPseeker (accessed on 1 July 2023)	[286]
Bismark	Alignment and analysis of DNAme data	https://github.com/FelixKrueger/Bismark (accessed on 1 July 2023)	[287]
BS Seeker	Alignment of bisulfite-treated reads to the reference genome	https://github.com/BSSeeker/BSseeker2 (accessed on 1 July 2023)	[288]
MethylKit	Analysis and visualization of DNAme data	https://github.com/al2na/methylKit (accessed on 1 July 2023)	[289]
Genomation	Visualization, annotation, and analysis of DNAme data	https://github.com/BIMSBbioinfo/genomation (accessed on 1 July 2023)	[290]
SnapATAC (Single Nucleus Analysis Pipeline for ATAC-seq)	scATAC-seq analysis (alignment of the read to a reference genome, quality control, peak calling, visualization, and clustering)	https://github.com/r3fang/SnapATAC (accessed on 1 July 2023)	[291]
Cellcano	Inference of cellular hierarchies of scATAC-seq data	https://marvinquiet.github.io/Cellcano/ (accessed on 1 July 2023)	[292]
Signac	Analysis and visualization of scATAC-seq data (peak calling, quality control, visualization, clustering, and integration with scRNA-seq data)	https://github.com/stuart-lab/signac (accessed on 1 July 2023)	[293]
EpiAnno	Analysis of scATAC-seq data	https://github.com/xy-chen16/EpiAnno (accessed on 1 July 2023)	[294]
**Enrichment Analysis**			
GSEA (gene set enrichment analysis)	Characterization of cellular functions as well as pathway enrichment analysis	https://www.gsea-msigdb.org/gsea/index.jsp (accessed on 1 July 2023)	[295]
IPA (Ingenuity Pathway Analysis)	Gene set analysis	https://digitalinsights.qiagen.com/products-overview/discovery-insights-portfolio/analysis-and-visualization/qiagen-ipa/ (accessed on 1 July 2023)	[296]
Enrichr	Integrative web-based tool for enrichment analysis	https://maayanlab.cloud/Enrichr/ (accessed on 19 August 2023)	[297,298,299]
FLAME	Integrative web-based tool for enrichment analysis	https://github.com/PavlopoulosLab/Flame (accessed on 19 August 2023)	[300]

## Supplementary Materials

The following supporting information can be downloaded at: https://www.mdpi.com/article/10.3390/cancers15174357/s1, Table S1: clinical trials of epigenetic modulators that have been or are being tested in PCa (retrieved from https://ClinicalTrials.gov, accessed on 31 May 2023).

## References

[1] Siegel R.L., Miller K.D., Fuchs H.E., Jemal A. (2022). Cancer Statistics, 2022. CA Cancer J. Clin..

[2] Gleason D.F., Tann M. (1977). Histologic Grading and Clinical Staging of Prostatic Carcinom.

[3] Delahunt B., Miller R.J., Srigley J.R., Evans A.J., Samaratunga H. (2012). Gleason Grading: Past, Present and Future. Histopathology.

[4] Schaeffer E.M., Srinivas S., Adra N., An Y., Barocas D., Bitting R., Bryce A., Chapin B., Cheng H.H., D’Amico A.V. (2022). NCCN GUIDELINES^®^ INSIGHTS: Prostate Cancer, Version 1.2023: Featured Updates to the NCCN Guidelines. JNCCN J. Natl. Compr. Cancer Netw..

[5] Mottet N., Bastian P., Bellmunt J., van den Bergh R., Bolla M., van Casteren N., Cornford P., Joniau S., Matveev V., van der Kwast T. (2020). EAU-EANM-ESTRO-ESUR-SIOG: Guidelines on Prostate Cancer. Eur. Assoc. Urol..

[6] Cornford P., van den Bergh R.C.N., Briers E., Van den Broeck T., Cumberbatch M.G., De Santis M., Fanti S., Fossati N., Gandaglia G., Gillessen S. (2021). EAU-EANM-ESTRO-ESUR-SIOG Guidelines on Prostate Cancer. Part II—2020 Update: Treatment of Relapsing and Metastatic Prostate Cancer [Formula Presented]. Eur. Urol..

[7] Mottet N., van den Bergh R.C.N., Briers E., Van den Broeck T., Cumberbatch M.G., De Santis M., Fanti S., Fossati N., Gandaglia G., Gillessen S. (2021). EAU-EANM-ESTRO-ESUR-SIOG Guidelines on Prostate Cancer—2020 Update. Part 1: Screening, Diagnosis, and Local Treatment with Curative Intent. Eur. Urol..

[8] Aparicio A., Logothetis C.J., Maity S.N. (2011). Understanding the Lethal Variant of Prostate Cancer: Power of Examining Extremes. Cancer Discov..

[9] Beltran H., Prandi D., Mosquera J.M., Benelli M., Puca L., Cyrta J., Marotz C., Giannopoulou E., Chakravarthi B.V.S.K., Varambally S. (2016). Divergent Clonal Evolution of Castration-Resistant Neuroendocrine Prostate Cancer. Nat. Med..

[10] Aparicio A.M., Harzstark A.L., Corn P.G., Wen S., Araujo J.C., Tu S.-M., Pagliaro L.C., Kim J., Millikan R.E., Ryan C. (2013). Platinum-Based Chemotherapy for Variant Castrate-Resistant Prostate Cancer. Clin. Cancer Res..

[11] Corn P.G., Heath E.I., Zurita A., Ramesh N., Xiao L., Sei E., Li-Ning-Tapia E., Tu S.M., Subudhi S.K., Wang J. (2019). Cabazitaxel plus Carboplatin for the Treatment of Men with Metastatic Castration-Resistant Prostate Cancers: A Randomised, Open-Label, Phase 1–2 Trial. Lancet Oncol..

[12] Quintanal-Villalonga Á., Chan J.M., Yu H.A., Pe’er D., Sawyers C.L., Sen T., Rudin C.M. (2020). Lineage Plasticity in Cancer: A Shared Pathway of Therapeutic Resistance. Nat. Rev. Clin. Oncol..

[13] Pérez-González A., Bévant K., Blanpain C. (2023). Cancer Cell Plasticity during Tumor Progression, Metastasis and Response to Therapy. Nat. Cancer.

[14] Davies A., Zoubeidi A., Beltran H., Selth L.A. (2023). The Transcriptional and Epigenetic Landscape of Cancer Cell Lineage Plasticity. Cancer Discov..

[15] Chaffer C.L., San Juan B.P., Lim E., Weinberg R.A. (2016). EMT, Cell Plasticity and Metastasis. Cancer Metastasis Rev..

[16] Esquer H., Zhou Q., Nemkov T., Abraham A.D., Rinaldetti S., Chen Y.C., Zhang X., Orman M.V., D’Alessandro A., Ferrer M. (2021). Isolating and Targeting the Real-Time Plasticity and Malignant Properties of Epithelial-Mesenchymal Transition in Cancer. Oncogene.

[17] Voon D.C., Huang R.Y., Jackson R.A., Thiery J.P. (2017). The EMT Spectrum and Therapeutic Opportunities. Mol. Oncol..

[18] Yuan S., Norgard R.J., Stanger B.Z. (2019). Cellular Plasticity in Cancer. Cancer Discov..

[19] Beltran H., Hruszkewycz A., Scher H.I., Hildesheim J., Isaacs J., Yu E.Y., Kelly K., Lin D., Dicker A., Arnold J. (2019). The Role of Lineage Plasticity in Prostate Cancer Therapy Resistance. Clin. Cancer Res..

[20] Terry S., Beltran H. (2014). The Many Faces of Neuroendocrine Differentiation in Prostate Cancer Progression. Front. Oncol..

[21] Espiritu S.M.G., Liu L.Y., Rubanova Y., Bhandari V., Holgersen E.M., Szyca L.M., Fox N.S., Chua M.L.K., Yamaguchi T.N., Heisler L.E. (2018). The Evolutionary Landscape of Localized Prostate Cancers Drives Clinical Aggression. Cell.

[22] Fraser M., Sabelnykova V.Y., Yamaguchi T.N., Heisler L.E., Livingstone J., Huang V., Shiah Y.J., Yousif F., Lin X., Masella A.P. (2017). Genomic Hallmarks of Localized, Non-Indolent Prostate Cancer. Nature.

[23] Wilt T.J., Ullman K.E., Linskens E.J., MacDonald R., Brasure M., Ester E., Nelson V.A., Saha J., Sultan S., Dahm P. (2021). Therapies for Clinically Localized Prostate Cancer: A Comparative Effectiveness Review. J. Urol..

[24] Zhao S.G., Chang S.L., Erho N., Yu M., Lehrer J., Alshalalfa M., Speers C., Cooperberg M.R., Kim W., Ryan C.J. (2017). Associations of Luminal and Basal Subtyping of Prostate Cancer With Prognosis and Response to Androgen Deprivation Therapy. JAMA Oncol..

[25] Blee A.M., He Y., Yang Y., Ye Z., Yan Y., Pan Y., Ma T., Dugdale J., Kuehn E., Kohli M. (2018). TMPrSS2-ERG Controls Luminal Epithelial Lineage and Antiandrogen Sensitivity in PTEN and TP53-Mutated Prostate Cancer. Clin. Cancer Res..

[26] Ku S.Y., Rosario S., Wang Y., Mu P., Seshadri M., Goodrich Z.W., Goodrich M.M., Labbé D.P., Gomez E.C., Wang J. (2017). Rb1 and Trp53 Cooperate to Suppress Prostate Cancer Lineage Plasticity, Metastasis, and Antiandrogen Resistance. Science.

[27] Mu P., Zhang Z., Benelli M., Karthaus W.R., Hoover E., Chen C.C., Wongvipat J., Ku S.Y., Gao D., Cao Z. (2017). SOX2 Promotes Lineage Plasticity and Antiandrogen Resistance in TP53-and RB1-Deficient Prostate Cancer. Science.

[28] Aparicio A.M., Shen L., Tapia E.L.N., Lu J.F., Chen H.C., Zhang J., Wu G., Wang X., Troncoso P., Corn P. (2016). Combined Tumor Suppressor Defects Characterize Clinically Defined Aggressive Variant Prostate Cancers. Clin. Cancer Res..

[29] Beltran H., Tomlins S., Aparicio A., Arora V., Rickman D., Ayala G., Huang J., True L., Gleave M.E., Soule H. (2014). Aggressive Variants of Castration-Resistant Prostate Cancer. Clin. Cancer Res..

[30] Matoso A., Epstein J.I. (2016). Grading of Prostate Cancer: Past, Present, and Future. Curr. Urol. Rep..

[31] Epstein J.I., Egevad L., Amin M.B., Delahunt B., Srigley J.R., Humphrey P.A., the Grading Committee (2016). The 2014 International Society of Urological Pathology (ISUP) Consensus Conference on Gleason Grading of Prostatic Carcinoma: Definition of Grading Patterns and Proposal for a New Grading System. Am. J. Surg. Pathol..

[32] Hussain M., Tangen C.M., Higano C., Schelhammer P.F., Faulkner J., Crawford E.D., Wilding G., Akdas A., Small E.J., Donnelly B. (2006). Absolute Prostate-Specific Antigen Value after Androgen Deprivation Is a Strong Independent Predictor of Survival in New Metastatic Prostate Cancer: Data from Southwest Oncology Group Trial 9346 (INT-0162). J. Clin. Oncol..

[33] Petrillo A., Fusco R., Setola S.V., Ronza F.M., Granata V., Petrillo M., Carone G., Sansone M., Franco R., Fulciniti F. (2014). Multiparametric MRI for Prostate Cancer Detection: Performance in Patients with Prostate-Specific Antigen Values between 2.5 and 10 Ng/ML. J. Magn. Reson. Imaging.

[34] Slatko B.E., Gardner A.F., Ausubel F.M. (2018). Overview of Next-Generation Sequencing Technologies. Curr. Protoc. Mol. Biol..

[35] Thankamony A.P., Subbalakshmi A.R., Jolly M.K., Nair R. (2021). Lineage Plasticity in Cancer: The Tale of a Skin-Walker. Cancers.

[36] Le Magnen C., Shen M.M., Abate-Shen C. (2018). Lineage Plasticity in Cancer Progression and Treatment. Annu. Rev. Cancer Biol..

[37] Zou M., Toivanen R., Mitrofanova A., Floch N., Hayati S., Sun Y., Le Magnen C., Chester D., Mostaghel E.A., Califano A. (2017). Transdifferentiation as a Mechanism of Treatment Resistance in a Mouse Model of Castration-Resistant Prostate Cancer. Cancer Discov..

[38] Haffner M.C., Mosbruger T., Esopi D.M., Fedor H., Heaphy C.M., Walker D.A., Adejola N., Gürel M., Hicks J., Meeker A.K. (2013). Tracking the Clonal Origin of Lethal Prostate Cancer. J. Clin. Investig..

[39] Waddington C.H. (2012). The Epigenotype. 1942. Int. J. Epidemiol..

[40] Lyko F. (2018). The DNA Methyltransferase Family: A Versatile Toolkit for Epigenetic Regulation. Nat. Rev. Genet..

[41] Sharma N.L., Massie C.E., Ramos-Montoya A., Zecchini V., Scott H.E., Lamb A.D., MacArthur S., Stark R., Warren A.Y., Mills I.G. (2013). The Androgen Receptor Induces a Distinct Transcriptional Program in Castration-Resistant Prostate Cancer in Man. Cancer Cell.

[42] Puca L., Bareja R., Prandi D., Shaw R., Benelli M., Karthaus W.R., Hess J., Sigouros M., Donoghue A., Kossai M. (2018). Patient Derived Organoids to Model Rare Prostate Cancer Phenotypes. Nat. Commun..

[43] Prasetyanti P.R., Medema J.P. (2017). Intra-Tumor Heterogeneity from a Cancer Stem Cell Perspective. Mol. Cancer.

[44] Davies A., Zoubeidi A., Selth L.A. (2020). The Epigenetic and Transcriptional Landscape of Neuroendocrine Prostate Cancer. Endocr. Relat. Cancer.

[45] Han H., Wang Y., Curto J., Gurrapu S., Laudato S., Rumandla A., Chakraborty G., Wang X., Chen H., Jiang Y. (2022). Mesenchymal and Stem-like Prostate Cancer Linked to Therapy-Induced Lineage Plasticity and Metastasis. Cell Rep..

[46] Takahashi K., Yamanaka S. (2006). Induction of Pluripotent Stem Cells from Mouse Embryonic and Adult Fibroblast Cultures by Defined Factors. Cell.

[47] Takahashi K., Yamanaka S. (2016). A Decade of Transcription Factor-Mediated Reprogramming to Pluripotency. Nat. Rev. Mol. Cell Biol..

[48] Paranjape A.N., Soundararajan R., Werden S.J., Joseph R., Taube J.H., Liu H., Rodriguez-Canales J., Sphyris N., Wistuba I., Miura N. (2016). Inhibition of FOXC2 Restores Epithelial Phenotype and Drug Sensitivity in Prostate Cancer Cells with Stem-Cell Properties. Oncogene.

[49] Soundararajan R., Paranjape A.N., Maity S., Aparicio A., Mani S.A. (2018). EMT, Stemness and Tumor Plasticity in Aggressive Variant Neuroendocrine Prostate Cancers. Biochim. Biophys. Acta-Rev. Cancer.

[50] Chan J.M., Zaidi S., Love J.R., Zhao J.L., Setty M., Wadosky K.M., Gopalan A., Choo Z.-N., Persad S., Choi J. (2022). Lineage Plasticity in Prostate Cancer Depends on JAK/STAT Inflammatory Signaling. Science.

[51] Brabletz T., Kalluri R., Nieto M.A., Weinberg R.A. (2018). EMT in Cancer. Nat. Rev. Cancer.

[52] Papanikolaou S., Vourda A., Syggelos S., Gyftopoulos K. (2021). Cell Plasticity and Prostate Cancer: The Role of Epithelial–Mesenchymal Transition in Tumor Progression, Invasion, Metastasis and Cancer Therapy Resistance. Cancers.

[53] Bakir B., Chiarella A.M., Pitarresi J.R., Rustgi A.K. (2020). EMT, MET, Plasticity, and Tumor Metastasis. Trends Cell Biol..

[54] Dardenne E., Beltran H., Benelli M., Gayvert K., Berger A., Puca L., Cyrta J., Sboner A., Noorzad Z., MacDonald T. (2016). N-Myc Induces an EZH2-Mediated Transcriptional Program Driving Neuroendocrine Prostate Cancer. Cancer Cell.

[55] Lee J.K., Phillips J.W., Smith B.A., Park J.W., Stoyanova T., McCaffrey E.F., Baertsch R., Sokolov A., Meyerowitz J.G., Mathis C. (2016). N-Myc Drives Neuroendocrine Prostate Cancer Initiated from Human Prostate Epithelial Cells. Cancer Cell.

[56] Berger A., Brady N.J., Bareja R., Robinson B., Conteduca V., Augello M.A., Puca L., Ahmed A., Dardenne E., Lu X. (2019). N-Myc-Mediated Epigenetic Reprogramming Drives Lineage Plasticity in Advanced Prostate Cancer. J. Clin. Investig..

[57] Beltran H., Oromendia C., Danila D.C., Montgomery B., Hoimes C., Szmulewitz R.Z., Vaishampayan U., Armstrong A.J., Stein M., Pinski J. (2019). A Phase II Trial of the Aurora Kinase a Inhibitor Alisertib for Patients with Castration-Resistant and Neuroendocrine Prostate Cancer: Efficacy and Biomarkers. Clin. Cancer Res..

[58] Jones D., Noble M., Wedge S.R., Robson C.N., Gaughan L. (2017). Aurora A Regulates Expression of AR-V7 in Models of Castrate Resistant Prostate Cancer. Sci. Rep..

[59] Beltran H., Rickman D.S., Park K., Chae S.S., Sboner A., MacDonald T.Y., Wang Y., Sheikh K.L., Terry S., Tagawa S.T. (2011). Molecular Characterization of Neuroendocrine Prostate Cancer and Identification of New Drug Targets. Cancer Discov..

[60] Ton A.T., Singh K., Morin H., Ban F., Leblanc E., Lee J., Lallous N., Cherkasov A. (2020). Dual-Inhibitors of N-Myc and AURKA as Potential Therapy for Neuroendocrine Prostate Cancer. Int. J. Mol. Sci..

[61] Heo Y.J., Hwa C., Lee G.H., Park J.M., An J.Y. (2021). Integrative Multi-Omics Approaches in Cancer Research: From Biological Networks to Clinical Subtypes. Mol. Cells.

[62] Rodriguez-Martin B., Alvarez E.G., Baez-Ortega A., Zamora J., Supek F., Demeulemeester J., Santamarina M., Ju Y.S., Temes J., Garcia-Souto D. (2020). Pan-Cancer Analysis of Whole Genomes Identifies Driver Rearrangements Promoted by LINE-1 Retrotransposition. Nat. Genet..

[63] Barbieri C.E., Bangma C.H., Bjartell A., Catto J.W.F., Culig Z., Grönberg H., Luo J., Visakorpi T., Rubin M.A. (2013). The Mutational Landscape of Prostate Cancer. Eur. Urol..

[64] Wang G., Zhao D., Spring D.J., DePinho R.A. (2018). Genetics and Biology of Prostate Cancer. Genes Dev..

[65] Farmer H., McCabe H., Lord C.J., Tutt A.H.J., Johnson D.A., Richardson T.B., Santarosa M., Dillon K.J., Hickson I., Knights C. (2005). Targeting the DNA Repair Defect in BRCA Mutant Cells as a Therapeutic Strategy. Nature.

[66] Feng F.Y., Brenner J.C., Hussain M., Chinnaiyan A.M. (2014). Molecular Pathways: Targeting ETS Gene Fusions in Cancer. Clin. Cancer Res. Off. J. Am. Assoc. Cancer Res..

[67] Grasso C.S., Wu Y.M., Robinson D.R., Cao X., Dhanasekaran S.M., Khan A.P., Quist M.J., Jing X., Lonigro R.J., Brenner J.C. (2012). The Mutational Landscape of Lethal Castration-Resistant Prostate Cancer. Nature.

[68] Sizemore G.M., Pitarresi J.R., Balakrishnan S., Ostrowski M.C. (2017). The ETS Family of Oncogenic Transcription Factors in Solid Tumours. Nat. Rev. Cancer.

[69] Kumar-Sinha C., Tomlins S.A., Chinnaiyan A.M. (2008). Recurrent Gene Fusions in Prostate Cancer. Nat. Rev. Cancer.

[70] Tomlins S.A., Rhodes D.R., Perner S., Dhanasekaran S.M., Mehra R., Sun X.W., Varambally S., Cao X., Tchinda J., Kuefer R. (2005). Recurrent Fusion of TMPRSS2 and ETS Transcription Factor Genes in Prostate Cancer. Science.

[71] Castro E., Goh C., Olmos D., Saunders E., Leongamornlert D., Tymrakiewicz M., Mahmud N., Dadaev T., Govindasami K., Guy M. (2013). Germline BRCA Mutations Are Associated with Higher Risk of Nodal Involvement, Distant Metastasis, and Poor Survival Outcomes in Prostate Cancer. J. Clin. Oncol..

[72] Pritchard C.C., Mateo J., Walsh M.F., De Sarkar N., Abida W., Beltran H., Garofalo A., Gulati R., Carreira S., Eeles R. (2016). Inherited DNA-Repair Gene Mutations in Men with Metastatic Prostate Cancer. N. Engl. J. Med..

[73] Oliva L., Lozano R., Llácer C., Aragón I., Pajares B.I., Sáez M.I., Herrera-Imbroda B., Montesa A., Hernández D., Villatoro R. (2021). Risk Prediction Tools Available for Germline BRCA1/2 Mutations Underperform in Prostate Cancer Patients. Eur. Urol. Oncol..

[74] Nyberg T., Frost D., Barrowdale D., Evans D.G., Bancroft E., Adlard J., Ahmed M., Barwell J., Brady A.F., Brewer C. (2020). Prostate Cancer Risks for Male BRCA1 [Formula Presented] and BRCA2 Mutation Carriers: A Prospective Cohort Study. Eur. Urol..

[75] Na R., Zheng S.L., Han M., Yu H., Jiang D., Shah S., Ewing C.M., Zhang L., Novakovic K., Petkewicz J. (2017). Germline Mutations in ATM and BRCA1/2 Distinguish Risk for Lethal and Indolent Prostate Cancer and Are Associated with Early Age at Death [Figure Presented]. Eur. Urol..

[76] Marshall C.H., Fu W., Wang H., Baras A.S., Lotan T.L., Antonarakis E.S. (2019). Prevalence of DNA Repair Gene Mutations in Localized Prostate Cancer According to Clinical and Pathologic Features: Association of Gleason Score and Tumor Stage. Prostate Cancer Prostatic Dis..

[77] Mohler J.L., Antonarakis E.S. (2019). NCCN Guidelines Updates: Management of Prostate Cancer. J. Natl. Compr. Canc. Netw..

[78] Antonarakis E.S., Shaukat F., Isaacsson Velho P., Kaur H., Shenderov E., Pardoll D.M., Lotan T.L. (2019). Clinical Features and Therapeutic Outcomes in Men with Advanced Prostate Cancer and DNA Mismatch Repair Gene Mutations. Eur. Urol..

[79] Yoshida T., Yaegashi H., Toriumi R., Kadomoto S., Iwamoto H., Izumi K., Kadono Y., Ikeda H., Mizokami A. (2022). Long Response Duration to Pembrolizumab in Metastatic, Castration-Resistant Prostate Cancer with Microsatellite Instability-High and Neuroendocrine Differentiation: A Case Report. Front. Oncol..

[80] Leongamornlert D., Mahmud N., Tymrakiewicz M., Saunders E., Dadaev T., Castro E., Goh C., Govindasami K., Guy M., O’Brien L. (2012). Germline BRCA1 Mutations Increase Prostate Cancer Risk. Br. J. Cancer.

[81] Nicolosi P., Ledet E., Yang S., Michalski S., Freschi B., O’Leary E., Esplin E.D., Nussbaum R.L., Sartor O. (2019). Prevalence of Germline Variants in Prostate Cancer and Implications for Current Genetic Testing Guidelines. JAMA Oncol..

[82] Blattner M., Lee D.J., O’Reilly C., Park K., MacDonald T.Y., Khani F., Turner K.R., Chiu Y.L., Wild P.J., Dolgalev I. (2014). SPOP Mutations in Prostate Cancer across Demographically Diverse Patient Cohorts. Neoplasia.

[83] Barbieri C.E., Baca S.C., Lawrence M.S., Demichelis F., Blattner M., Theurillat J.P., White T.A., Stojanov P., Van Allen E., Stransky N. (2012). Exome Sequencing Identifies Recurrent SPOP, FOXA1 and MED12 Mutations in Prostate Cancer. Nat. Genet..

[84] Nakazawa M., Fang M., Issacsson Velho P., Antonarakis E.S. (2021). SPOP Mutations in Prostate Cancer: Clinical and Genomic Features. J. Clin. Oncol..

[85] Dai X., Gan W., Li X., Wang S., Zhang W., Huang L., Liu S., Zhong Q., Guo J., Zhang J. (2017). Prostate Cancer-Associated SPOP Mutations Confer Resistance to BET Inhibitors through Stabilization of BRD4. Nat. Med..

[86] de Bono J., Mateo J., Fizazi K., Saad F., Shore N., Sandhu S., Chi K.N., Sartor O., Agarwal N., Olmos D. (2020). Olaparib for Metastatic Castration-Resistant Prostate Cancer. N. Engl. J. Med..

[87] Oh M., Alkhushaym N., Fallatah S., Althagafi A., Aljadeed R., Alsowaida Y., Jeter J., Martin J.R., Babiker H.M., McBride A. (2019). The Association of BRCA1 and BRCA2 Mutations with Prostate Cancer Risk, Frequency, and Mortality: A Meta-Analysis. Prostate.

[88] Jamaspishvili T., Berman D.M., Ross A.E., Scher H.I., De Marzo A.M., Squire J.A., Lotan T.L. (2018). Clinical Implications of PTEN Loss in Prostate Cancer. Nat. Rev. Urol..

[89] Thangavel C., Boopathi E., Liu Y., Haber A., Ertel A., Bhardwaj A., Addya S., Williams N., Ciment S.J., Cotzia P. (2017). RB Loss Promotes Prostate Cancer Metastasis. Cancer Res..

[90] Aparicio A., Den R.B., Knudsen K.E. (2011). Time to Stratify? The Retinoblastoma Protein in Castrate-Resistant Prostate Cancer. Nat. Rev. Urol..

[91] Han W., Liu M., Han D., Li M., Toure A.A., Wang Z., Besschetnova A., Patalano S., Macoska J.A., Gao S. (2022). RB1 Loss in Castration-Resistant Prostate Cancer Confers Vulnerability to LSD1 Inhibition. Oncogene.

[92] Mandigo A.C., McNair C., Ku K., Pang A., Guan Y.F., Holst J., Brown M., Kelly W.K., Knudsen K.E. (2020). Molecular Underpinnings of RB Status as a Biomarker of Poor Outcome in Advanced Prostate Cancer. J. Clin. Oncol..

[93] Quigley D.A., Dang H.X., Zhao S.G., Lloyd P., Aggarwal R., Alumkal J.J., Foye A., Kothari V., Perry M.D., Bailey A.M. (2018). Genomic Hallmarks and Structural Variation in Metastatic Prostate Cancer. Cell.

[94] Robinson D., Van Allen E.M., Wu Y.M., Schultz N., Lonigro R.J., Mosquera J.M., Montgomery B., Taplin M.E., Pritchard C.C., Attard G. (2015). Integrative Clinical Genomics of Advanced Prostate Cancer. Cell.

[95] National Comprehensive Cancer Network Prostate Cancer NCCN Guidelines (Version 3.2023). https://www.nccn.org/guidelines/recently-published-guidelines.

[96] Tan H.-L., Sood A., Rahimi H.A., Wang W., Gupta N., Hicks J., Mosier S., Gocke C.D., Epstein J.I., Netto G.J. (2014). Rb Loss Is Characteristic of Prostatic Small Cell Neuroendocrine Carcinoma. Clin. Cancer Res..

[97] Gurevich L.E., Bondarenko E.V., Vasyukova O.A., Mikhaleva L.M. (2021). The Role of Yamanaka Cocktail Transcription Factors (OCT4, SOX2, KLF4, c-Myc) in the Differentiation of Somatic Cells, Their Malignant Transformation, and Tumor Progression. Clin. Exp. Morphol..

[98] Yoshimoto M., Cutz J.C., Nuin P.A.S., Joshua A.M., Bayani J., Evans A.J., Zielenska M., Squire J.A. (2006). Interphase FISH Analysis of PTEN in Histologic Sections Shows Genomic Deletions in 68% of Primary Prostate Cancer and 23% of High-Grade Prostatic Intra-Epithelial Neoplasias. Cancer Genet. Cytogenet..

[99] Myint Z.W., Allison D.B., Ellis C.S. (2021). A Case Report of Metastatic Castration-Resistant Prostate Cancer Harboring a PTEN Loss. Front. Oncol..

[100] Leinonen K.A., Saramäki O.R., Furusato B., Kimura T., Takahashi H., Egawa S., Suzuki H., Keiger K., Hahm S.H., Isaacs W.B. (2013). Loss of PTEN Is Associated with Aggressive Behavior in ERG-Positive Prostate Cancer. Cancer Epidemiol. Biomarkers Prev..

[101] Abeshouse A., Ahn J., Akbani R., Ally A., Amin S., Andry C.D., Annala M., Aprikian A., Armenia J., Arora A. (2015). The Molecular Taxonomy of Primary Prostate Cancer. Cell.

[102] Choudhury A.D. (2022). PTEN-PI3K Pathway Alterations in Advanced Prostate Cancer and Clinical Implications. Prostate.

[103] Acikalin Coskun K., Tutar M., Al M., Gok Yurttas A., Cansu Abay E., Yurekli N., Yeman Kiyak B., Ucar Cifci K., Tutar Y. (2022). Role of P53 in Human Cancers. p53—A Guardian of the Genome and Beyond.

[104] Ecke T.H., Schlechte H.H., Schiemenz K., Sachs M.D., Lenk S.V., Rudolph B.D., Loening S.A. (2010). TP53 Gene Mutations in Prostate Cancer Progression. Anticancer. Res..

[105] Teroerde M., Nientiedt C., Duensing A., Hohenfellner M., Stenzinger A., Duensing S. (2021). Revisiting the Role of P53 in Prostate Cancer. Prostate Cancer.

[106] Hientz K., Mohr A., Bhakta-Guha D., Efferth T. (2017). The Role of P53 in Cancer Drug Resistance and Targeted Chemotherapy. Oncotarget.

[107] Kim S., An S.S.A. (2016). Role of P53 Isoforms and Aggregations in Cancer. Medicine.

[108] Ozaki T., Nakagawara A. (2011). Role of P53 in Cell Death and Human Cancers. Cancers.

[109] Busuttil R.A., Zapparoli G.V., Haupt S., Fennell C., Wong S.Q., Pang J.M.B., Takeno E.A., Mitchell C., Di Costanzo N., Fox S. (2014). Role of P53 in the Progression of Gastric Cancer. Oncotarget.

[110] Antonarakis E.S., Lu C., Wang H., Luber B., Nakazawa M., Roeser J.C., Chen Y., Mohammad T.A., Chen Y., Fedor H.L. (2014). AR-V7 and Resistance to Enzalutamide and Abiraterone in Prostate Cancer. N. Engl. J. Med..

[111] Azad A., Todenhöfer T., Stewart C., Gao J., Eigl B.J., Black P.C., Joshua A.M., Chi K.N. (2016). Correlation of AR-V7 Expression in Whole Blood with Efficacy of Abiraterone Acetate (ABI) in Metastatic Castration-Resistant Prostate Cancer (MCRPC) Patients (Pts). J. Clin. Oncol..

[112] Antonarakis E.S., Lu C., Luber B., Wang H., Chen Y., Zhu Y., Silberstein J.L., Taylor M.N., Maughan B.L., Paller C.J. (2016). AR-V7 and Efficacy of Abiraterone (Abi) and Enzalutamide (Enza) in Castration-Resistant Prostate Cancer (CRPC): Expanded Analysis of the Johns Hopkins Cohort. J. Clin. Oncol..

[113] Jerónimo C., Usadel H., Henrique R., Silva C., Oliveira J., Lopes C., Sidransky D. (2002). Quantitative GSTP1 Hypermethylation in Bodily Fluids of Patients with Prostate Cancer. Urology.

[114] Jamshidian F., Akbari M.T., Noormohammadi Z., Nourozi M.R., Pourmand G.R. (2016). CpG Islands Hypermethylatioin the Promoter Region of GSTP1 Gene in Cell-Free DNA as a Noninvasive Biomarker for Detecting Prostate Cancer. Biochem. Cell. Arch..

[115] Friedemann M., Horn F., Gutewort K., Tautz L., Jandeck C., Bechmann N., Sukocheva O., Wirth M.P., Fuessel S., Menschikowski M. (2021). Increased Sensitivity of Detection of Rassf1a and Gstp1 Dna Fragments in Serum of Prostate Cancer Patients: Optimisation of Diagnostics Using Obbpa-Ddpcr. Cancers.

[116] Pećina-Šlaus N. (2003). Tumor Suppressor Gene E-Cadherin and Its Role in Normal and Malignant Cells. Cancer Cell Int..

[117] Quinn D.I., Henshall S.M., Sutherland R.L. (2005). Molecular Markers of Prostate Cancer Outcome. Eur. J. Cancer.

[118] Ouhtit A., Rizeq B., Saleh H.A., Rahman M.D.M., Zayed H. (2018). Novel CD44-Downstream Signaling Pathways Mediating Breast Tumor Invasion. Int. J. Biol. Sci..

[119] Sugiura M., Sato H., Kanesaka M., Imamura Y., Sakamoto S., Ichikawa T., Kaneda A. (2021). Epigenetic Modifications in Prostate Cancer. Int. J. Urol..

[120] Moison C., Assemat F., Daunay A., Tost J., Guieysse-Peugeot A.L., Arimondo P.B. (2014). Synergistic Chromatin Repression of the Tumor Suppressor Gene RARB in Human Prostate Cancers. Epigenetics.

[121] Kang G.H., Lee S., Lee H.J., Hwang K.S. (2004). Aberrant CpG Island Hypermethylation of Multiple Genes in Prostate Cancer and Prostatic Intraepithelial Neoplasia. J. Pathol..

[122] Liu L., Yoon J.H., Dammann R., Pfeifer G.P. (2002). Frequent Hypermethylation of the Rassf1a Gene in Prostate Cancer. Oncogene.

[123] Yaqinuddin A., Qureshi S.A., Pervez S., Bashir M.U., Nazir R., Abbas F. (2013). Frequent DNA Hypermethylation at the RASSF1A and APC Gene Loci in Prostate Cancer Patients of Pakistani Origin. ISRN Urol..

[124] Maruyama R., Toyooka S., Toyooka K.O., Virmani A.K., Zöchbauer-Müller S., Farinas A.J., Minna J.D., McConnell J., Frenkel E.P., Gazdar A.F. (2002). Aberrant Promoter Methylation Profile of Prostate Cancers and Its Relationship to Clinicopathological Features. Clin. Cancer Res..

[125] Woodson K., Hayes R., Wideroff L., Villaruz L., Tangrea J. (2003). Hypermethylation of GSTP1, CD44, and E-Cadherin Genes in Prostate Cancer among US Blacks and Whites. Prostate.

[126] Uhl B., Gevensleben H., Tolkach Y., Sailer V., Majores M., Jung M., Meller S., Stein J., Ellinger J., Dietrich D. (2017). PITX2 DNA Methylation as Biomarker for Individualized Risk Assessment of Prostate Cancer in Core Biopsies. J. Mol. Diagn..

[127] Steiner I., Jung K., Schatz P., Horns T., Wittschieber D., Lein M., Dietel M., Erbersdobler A. (2010). Gene Promoter Methylation and Its Potential Relevance in Early Prostate Cancer Diagnosis. Pathobiology.

[128] Gurioli G., Martignano F., Salvi S., Costantini M., Gunelli R., Casadio V. (2018). GSTP1 Methylation in Cancer: A Liquid Biopsy Biomarker?. Clin. Chem. Lab. Med..

[129] Fiano V., Zugna D., Grasso C., Trevisan M., Delsedime L., Molinaro L., Gillio-Tos A., Merletti F., Richiardi L. (2017). LINE-1 Methylation Status in Prostate Cancer and Non-Neoplastic Tissue Adjacent to Tumor in Association with Mortality. Epigenetics.

[130] Zelic R., Fiano V., Zugna D., Grasso C., Delsedime L., Daniele L., Galliano D., Pettersson A., Gillio-Tos A., Merletti F. (2016). Global Hypomethylation (LINE-1) and Gene-Specific Hypermethylation (GSTP1) on Initial Negative Prostate Biopsy as Markers of Prostate Cancer on a Rebiopsy. Clin. Cancer Res..

[131] Kleb B., Estécio M.R.H., Zhang J., Tzelepi V., Chung W., Jelinek J., Navone N.M., Tahir S., Marquez V.E., Issa J.P. (2016). Differentially Methylated Genes and Androgen Receptor Re-Expression in Small Cell Prostate Carcinomas. Epigenetics.

[132] Yamada Y., Beltran H. (2021). Clinical and Biological Features of Neuroendocrine Prostate Cancer. Curr. Oncol. Rep..

[133] Gautam N., Kaur M., Kaur S. (2021). Structural Assembly of Polycomb Group Protein and Insight of EZH2 in Cancer Progression: A Review. J. Cancer Res. Ther..

[134] Hoffmann M.J., Engers R., Florl A.R., Otte A.P., Müller M., Schulz W.A. (2007). Expression Changes in EZH2, but Not in BMI-1, SIRT1, DNMT1 or DNMT3B, Are Associated with DNA Methylation Changes in Prostate Cancer. Cancer Biol. Ther..

[135] Gu X., Gao X.S., Bai Y., Cui M., Xiong W., Han L., Guo W., Xie M., Peng C., Su M. (2016). EZH2 Overexpression as a Biomarker of Poor Prognosis in Prostate Cancer. Int. J. Clin. Exp. Med..

[136] Sehrawat A., Gao L., Wang Y., Bankhead A., McWeeney S.K., King C.J., Schwartzman J., Urrutia J., Bisson W.H., Coleman D.J. (2018). LSD1 Activates a Lethal Prostate Cancer Gene Network Independently of Its Demethylase Function. Proc. Natl. Acad. Sci. USA.

[137] Rudolph T., Beuch S., Reuter G. (2013). Lysine-Specific Histone Demethylase LSD1 and the Dynamic Control of Chromatin. Biol. Chem..

[138] Tzelepi V., Zhang J., Lu J.-F., Kleb B., Wu G., Wan X., Hoang A., Efstathiou E., Sircar K., Navone N.M. (2012). Modeling a Lethal Prostate Cancer Variant with Small-Cell Carcinoma Features. Clin. Cancer Res..

[139] Anselmino N., Labanca E., Song X., Yang J., Shepherd P.D.A., Dong J., Kundra R., Schultz N., Zhang J., Araujo J.C. (2022). Integrative Analysis of the MD Anderson Prostate Cancer Patient-Derived Xenograft Series (MDA PCa PDX). bioRxiv.

[140] Hong H., Kao C., Jeng M.H., Eble J.N., Koch M.O., Gardner T.A., Zhang S., Li L., Pan C.X., Hu Z. (2004). Aberrant Expression of CARM1, a Transcriptional Coactivator of Androgen Receptor, in the Development of Prostate Carcinoma and Androgen-Independent Status. Cancer.

[141] Grypari I.M., Logotheti S., Zolota V., Troncoso P., Efstathiou E., Bravou V., Melachrinou M., Logothetis C., Tzelepi V. (2021). The Protein Arginine Methyltransferases (PRMTs) PRMT1 and CARM1 as Candidate Epigenetic Drivers in Prostate Cancer Progression. Medicine.

[142] Raposo A.E., Piller S.C. (2018). Protein Arginine Methylation: An Emerging Regulator of the Cell Cycle. Cell Div..

[143] Dawson M.A., Kouzarides T. (2012). Cancer Epigenetics: From Mechanism to Therapy. Cell.

[144] Chen Q.W., Zhu X.Y., Li Y.Y., Meng Z.Q. (2014). Epigenetic Regulation and Cancer (Review). Oncol. Rep..

[145] Skourti E., Dhillon P. (2022). Cancer Epigenetics: Promises and Pitfalls for Cancer Therapy. FEBS J..

[146] Flintoft L. (2008). Epigenetics: DNA Methylation Gets Dynamic. Nat. Rev. Genet..

[147] Jerónimo C., Bastian P.J., Bjartell A., Carbone G.M., Catto J.W.F., Clark S.J., Henrique R., Nelson W.G., Shariat S.F. (2011). Epigenetics in Prostate Cancer: Biologic and Clinical Relevance. Eur. Urol..

[148] Jones P.A. (2012). Functions of DNA Methylation: Islands, Start Sites, Gene Bodies and Beyond. Nat. Rev. Genet..

[149] Patel P.G., Wessel T., Kawashima A., Okello J.B.A., Jamaspishvili T., Guérard K.P., Lee L., Lee A.Y.W., How N.E., Dion D. (2019). A Three-Gene DNA Methylation Biomarker Accurately Classifies Early Stage Prostate Cancer. Prostate.

[150] Luan Z.M., Zhang H., Qu X.L. (2016). Prediction Efficiency of PITX2 DNA Methylation for Prostate Cancer Survival. Genet. Mol. Res..

[151] Li J.Z., Zhang Y., Wen B., Li M., Wang Y.J. (2015). Ability of PITX2 Methylation to Predict Survival in Patients with Prostate Cancer. Onco. Targets. Ther..

[152] Tzelepi V., Logotheti S., Efstathiou E., Troncoso P., Aparicio A., Sakellakis M., Hoang A., Perimenis P., Melachrinou M., Logothetis C. (2020). Epigenetics and Prostate Cancer: Defining the Timing of DNA Methyltransferase Deregulation during Prostate Cancer Progression. Pathology.

[153] Chen S., Petricca J., Ye W., Guan J., Zeng Y., Cheng N., Gong L., Shen S.Y., Hua J.T., Crumbaker M. (2022). The Cell-Free DNA Methylome Captures Distinctions between Localized and Metastatic Prostate Tumors. Nat. Commun..

[154] Loyfer N., Magenheim J., Peretz A., Cann G., Bredno J., Klochendler A., Fox-Fisher I., Shabi-Porat S., Hecht M., Pelet T. (2023). A DNA Methylation Atlas of Normal Human Cell Types. Nature.

[155] Steffan J.J., Koul S., Meacham R.B., Koul H.K. (2012). The Transcription Factor SPDEF Suppresses Prostate Tumor Metastasis. J. Biol. Chem..

[156] Niemeyer C.M., Flotho C., Lipka D.B., Stary J., Rössig C., Baruchel A., Klingebiel T., Micalizzi C., Michel G., Nysom K. (2021). Response to Upfront Azacitidine in Juvenile Myelomonocytic Leukemia in the AZA-JMML-001 Trial. Blood Adv..

[157] Niemeyer C.M., Flotho C., Lipka D.B., Starý J., Rössig C., Baruchel A., Klingebiel T., Micalizzi C., Michel G., Nysom K. (2019). Upfront Azacitidine (AZA) in Juvenile Myelomonocytic Leukemia (JMML): Interim Analysis of the Prospective AZA-JMML-001 Study. J. Clin. Oncol..

[158] Janssen A., Breuer G.A., Brinkman E.K., Van Der Meulen A.I., Borden S.V., Van Steense B., Bindra R.S., Larocque J.R., Karpen G.H. (2016). A Single Double-Strand Break System Reveals Repair Dynamics and Mechanisms in Heterochromatin and Euchromatin. Genes Dev..

[159] Fischle W. (2012). One, Two, Three: How Histone Methylation Is Read. Epigenomics.

[160] Kouzarides T. (2007). Chromatin Modifications and Their Function. Cell.

[161] Bernstein B.E., Meissner A., Lander E.S. (2007). The Mammalian Epigenome. Cell.

[162] Khare S.P., Habib F., Sharma R., Gadewal N., Gupta S., Galande S. (2012). HIstome—A Relational Knowledgebase of Human Histone Proteins and Histone Modifying Enzymes. Nucleic Acids Res..

[163] Haberland M., Montgomery R.L., Olson E.N. (2009). The Many Roles of Histone Deacetylases in Development and Physiology: Implications for Disease and Therapy. Nat. Rev. Genet..

[164] Shi Y. (2007). Histone Lysine Demethylases: Emerging Roles in Development, Physiology and Disease. Nat. Rev. Genet..

[165] Sharma S., Kelly T.K., Jones P.A. (2009). Epigenetics in Cancer. Carcinogenesis.

[166] Loenarz C., Ge W., Coleman M.L., Rose N.R., Cooper C.D.O., Klose R.J., Ratcliffe P.J., Schofield C.J. (2010). PHF8, a Gene Associated with Cleft Lip/Palate and Mental Retardation, Encodes for an Nepsilon-Dimethyl Lysine Demethylase. Hum. Mol. Genet..

[167] Cao R., Wang L., Wang H., Xia L., Erdjument-Bromage H., Tempst P., Jones R.S., Zhang Y. (2002). Role of Histone H3 Lysine 27 Methylation in Polycomb-Group Silencing. Science.

[168] Rana Z., Diermeier S., Hanif M., Rosengren R.J. (2020). Understanding Failure and Improving Treatment Using HDAC Inhibitors for Prostate Cancer. Biomedicines.

[169] Davies A., Nouruzi S., Ganguli D., Namekawa T., Thaper D., Linder S., Karaoğlanoğlu F., Omur M.E., Kim S., Kobelev M. (2021). An Androgen Receptor Switch Underlies Lineage Infidelity in Treatment-Resistant Prostate Cancer. Nat. Cell Biol..

[170] Storck W.K., May A.M., Westbrook T.C., Duan Z., Morrissey C., Yates J.A., Alumkal J.J. (2022). The Role of Epigenetic Change in Therapy-Induced Neuroendocrine Prostate Cancer Lineage Plasticity. Front. Endocrinol..

[171] He L., Hannon G.J. (2004). Correction: MicroRNAs: Small RNAs with a Big Role in Gene Regulation. Nat. Rev. Genet..

[172] Wang X.W., Hu L.F., Hao J., Liao L.Q., Chiu Y.T., Shi M., Wang Y. (2019). A MicroRNA-Inducible CRISPR–Cas9 Platform Serves as a MicroRNA Sensor and Cell-Type-Specific Genome Regulation Tool. Nat. Cell Biol..

[173] Misawa A., Takayama K.I., Inoue S. (2017). Long Non-Coding RNAs and Prostate Cancer. Cancer Sci..

[174] Ha M., Kim V.N. (2014). Regulation of MicroRNA Biogenesis. Nat. Rev. Mol. Cell Biol..

[175] Prensner J.R., Chinnaiyan A.M. (2011). The Emergence of LncRNAs in Cancer Biology. Cancer Discov..

[176] Smolle M.A., Bauernhofer T., Pummer K., Calin G.A., Pichler M. (2017). Current Insights into Long Non-Coding RNAs (LncRNAs) in Prostate Cancer. Int. J. Mol. Sci..

[177] Zhang A., Zhang J., Kaipainen A., Lucas J.M., Yang H. (2016). Long Non-Coding RNA: A Newly Deciphered “Code” in Prostate Cancer. Cancer Lett..

[178] An C., Wang I., Li X., Xia R., Deng F. (2022). Long Non-Coding RNA in Prostate Cancer. Am. J. Clin. Exp. Urol..

[179] Pickard M.R., Mourtada-Maarabouni M., Williams G.T. (2013). Long Non-Coding RNA GAS5 Regulates Apoptosis in Prostate Cancer Cell Lines. Biochim. Biophys. Acta-Mol. Basis Dis..

[180] Gong X., Ning B. (2020). Five LncRNAs Associated With Prostate Cancer Prognosis Identified by Coexpression Network Analysis. Technol. Cancer Res. Treat..

[181] Xiong T., Li J., Chen F., Zhang F. (2019). PCAT-1: A Novel Oncogenic Long Non-Coding RNA in Human Cancers. Int. J. Biol. Sci..

[182] Yang Z., Zhao S., Zhou X., Zhao H., Jiang X. (2019). PCAT-1: A Pivotal Oncogenic Long Non-Coding RNA in Human Cancers. Biomed. Pharmacother..

[183] Prensner J.R., Iyer M.K., Balbin O.A., Dhanasekaran S.M., Cao Q., Brenner J.C., Laxman B., Asangani I., Grasso C., Kominsky H.D. (2012). Transcriptome Sequencing Identifies PCAT-1, a Novel LincRNA Implicated in Prostate Cancer Progression. Nat. Biotechnol..

[184] Prensner J.R., Chen W., Han S., Iyer M.K., Cao Q., Kothari V., Evans J.R., Knudsen K.E., Paulsen M.T., Ljungman M. (2014). The Long Non-Coding RNA PCAT-1 Promotes Prostate Cancer Cell Proliferation through CMyc. Neoplasia.

[185] White N.M., Zhao S.G., Zhang J., Rozycki E.B., Dang H.X., McFadden S.D., Eteleeb A.M., Alshalalfa M., Vergara I.A., Erho N. (2017). Multi-Institutional Analysis Shows That Low PCAT-14 Expression Associates with Poor Outcomes in Prostate Cancer. Eur. Urol..

[186] Singh N., Ramnarine V.R., Song J.H., Pandey R., Padi S.K.R., Nouri M., Olive V., Kobelev M., Okumura K., McCarthy D. (2021). The Long Noncoding RNA H19 Regulates Tumor Plasticity in Neuroendocrine Prostate Cancer. Nat. Commun..

[187] Bonci D., Coppola V., Patrizii M., Addario A., Cannistraci A., Francescangeli F., Pecci R., Muto G., Collura D., Bedini R. (2016). A MicroRNA Code for Prostate Cancer Metastasis. Oncogene.

[188] Chiam K., Ricciardelli C., Bianco-Miotto T. (2014). Epigenetic Biomarkers in Prostate Cancer: Current and Future Uses. Cancer Lett..

[189] Walter B.A., Valera V.A., Pinto P.A., Merino M.J. (2013). Comprehensive MicroRNA Profiling of Prostate Cancer. J. Cancer.

[190] Folini M., Gandellini P., Longoni N., Profumo V., Callari M., Pennati M., Colecchia M., Supino R., Veneroni S., Salvioni R. (2010). MiR-21: An Oncomir on Strike in Prostate Cancer. Mol. Cancer.

[191] Ghorbanmehr N., Gharbi S., Korsching E., Tavallaei M., Einollahi B., Mowla S.J. (2019). MiR-21-5p, MiR-141-3p, and MiR-205-5p Levels in Urine—Promising Biomarkers for the Identification of Prostate and Bladder Cancer. Prostate.

[192] Stafford M.Y.C., Willoughby C.E., Walsh C.P., McKenna D.J. (2022). Prognostic Value of MiR-21 for Prostate Cancer: A Systematic Review and Meta-Analysis. Biosci. Rep..

[193] Cheng C.Y., Hwang C.I., Corney D.C., Flesken-Nikitin A., Jiang L., Öner G.M., Munroe R.J., Schimenti J.C., Hermeking H., Nikitin A.Y. (2014). MiR-34 Cooperates with P53 in Suppression of Prostate Cancer by Joint Regulation of Stem Cell Compartment. Cell Rep..

[194] Oh-Hohenhorst S.J., Lange T. (2021). Role of Metastasis-Related Micrornas in Prostate Cancer Progression and Treatment. Cancers.

[195] Zhao Z., Weickmann S., Jung M., Lein M., Kilic E., Stephan C., Erbersdobler A., Fendler A., Jung K. (2019). A Novel Predictor Tool of Biochemical Recurrence after Radical Prostatectomy Based on a Five-MicroRNA Tissue Signature. Cancers.

[196] Liu F., Fan Y., Ou L., Li T., Fan J., Duan L., Yang J., Luo C., Wu X. (2020). CircHIPK3 Facilitates the G2/M Transition in Prostate Cancer Cells by Sponging MiR-338-3p. Onco. Targets. Ther..

[197] Chen D., Lu X., Yang F., Xing N. (2019). Circular RNA CircHIPK3 Promotes Cell Proliferation and Invasion of Prostate Cancer by Sponging MiR-193a-3p and Regulating MCL1 Expression. Cancer Manag. Res..

[198] Liu D.C., Song L.L., Li X.Z., Liang Q., Zhang Z.G., Han C.H. (2021). Circular RNA CircHIPK3 Modulates Prostate Cancer Progression via Targeting MiR-448/MTDH Signaling. Clin. Transl. Oncol..

[199] Cai C., Zhi Y., Wang K., Zhang P., Ji Z., Xie C., Sun F. (2019). CircHIPK3 Overexpression Accelerates the Proliferation and Invasion of Prostate Cancer Cells through Regulating MiRNA-338-3p. Onco. Targets. Ther..

[200] Xie X., Sun F.K., Huang X., Wang C.H., Dai J., Zhao J.P., Fang C., He W. (2021). A Circular RNA, CircSMARCA5, Inhibits Prostate Cancer Proliferative, Migrative, and Invasive Capabilities via the MiR-181b-5p/MiR-17-3p-TIMP3 Axis. Aging.

[201] Yi C., Wan X., Zhang Y., Fu F., Zhao C., Qin R., Wu H., Li Y., Huang Y. (2018). SNORA42 Enhances Prostate Cancer Cell Viability, Migration and EMT and Is Correlated with Prostate Cancer Poor Prognosis. Int. J. Biochem. Cell Biol..

[202] Zhang L., Meng X., Pan C., Qu F., Gan W., Xiang Z., Han X., Li D. (2020). PiR-31470 Epigenetically Suppresses the Expression of Glutathione S-Transferase Pi 1 in Prostate Cancer via DNA Methylation. Cell. Signal..

[203] Zhang L., Meng X., Li D., Han X. (2020). PiR-001773 and PiR-017184 Promote Prostate Cancer Progression by Interacting with PCDH9. Cell. Signal..

[204] Visser W.C.H., de Jong H., Melchers W.J.G., Mulders P.F.A., Schalken J.A. (2020). Commercialized Blood-, Urinary-and Tissue-Based Biomarker Tests for Prostate Cancer Diagnosis and Prognosis. Cancers.

[205] Salami S.S., Hovelson D.H., Kaplan J.B., Mathieu R., Udager A.M., Curci N.E., Lee M., Plouffe K.R., de la Vega L.L., Susani M. (2018). Transcriptomic Heterogeneity in Multifocal Prostate Cancer. JCI Insight.

[206] Wei L., Wang J., Lampert E., Schlanger S., DePriest A.D., Hu Q., Gomez E.C., Murakam M., Glenn S.T., Conroy J. (2017). Intratumoral and Intertumoral Genomic Heterogeneity of Multifocal Localized Prostate Cancer Impacts Molecular Classifications and Genomic Prognosticators. Eur. Urol..

[207] Chen S., Zhu G., Yang Y., Wang F., Xiao Y.T., Zhang N., Bian X., Zhu Y., Yu Y., Liu F. (2021). Single-Cell Analysis Reveals Transcriptomic Remodellings in Distinct Cell Types That Contribute to Human Prostate Cancer Progression. Nat. Cell Biol..

[208] Spratt D.E., Alshalalfa M., Fishbane N., Weiner A.B., Mehra R., Mahal B.A., Lehrer J., Liu Y., Zhao S.G., Speers C. (2019). Transcriptomic Heterogeneity of Androgen Receptor Activity Defines a de Novo Low AR-Active Subclass in Treatment Naïve Primary Prostate Cancer. Clin. Cancer Res..

[209] Sutera P.A., Shetty A.C., Hakansson A., Van der Eecken K., Song Y., Liu Y., Chang J., Fonteyne V., Mendes A.A., Lumen N. (2023). Transcriptomic and Clinical Heterogeneity of Metastatic Disease Timing within Metastatic Castration-Sensitive Prostate Cancer. Ann. Oncol..

[210] Han H., Lee H.H., Choi K., Moon Y.J., Heo J.E., Ham W.S., Jang W.S., Rha K.H., Cho N.H., Giancotti F.G. (2021). Prostate Epithelial Genes Define Therapy-Relevant Prostate Cancer Molecular Subtype. Prostate Cancer Prostatic Dis..

[211] Cuzick J.M., Fisher G., Berney D., Mesher D., Møller H., Reid J.E., Gutin A., Lanchbury J.S., Stone S. (2011). Prognostic Value of a 46-Gene Cell Cycle Progression (CCP) RNA Signature for Prostate Cancer Death in a Conservatively Managed Watchful Waiting Needle Biopsy Cohort. J. Clin. Oncol..

[212] Erho N., Crisan A., Vergara I.A., Mitra A.P., Ghadessi M., Buerki C., Bergstralh E.J., Kollmeyer T., Fink S., Haddad Z. (2013). Discovery and Validation of a Prostate Cancer Genomic Classifier That Predicts Early Metastasis Following Radical Prostatectomy. PLoS ONE.

[213] Klein E.A., Cooperberg M.R., Magi-Galluzzi C., Simko J.P., Falzarano S.M., Maddala T., Chan J.M., Li J., Cowan J.E., Tsiatis A.C. (2014). A 17-Gene Assay to Predict Prostate Cancer Aggressiveness in the Context of Gleason Grade Heterogeneity, Tumor Multifocality, and Biopsy Undersampling. Eur. Urol..

[214] Ståhl P.L., Salmén F., Vickovic S., Lundmark A., Navarro J.F., Magnusson J., Giacomello S., Asp M., Westholm J.O., Huss M. (2016). Visualization and Analysis of Gene Expression in Tissue Sections by Spatial Transcriptomics. Science.

[215] Yu Q., Jiang M., Wu L. (2022). Spatial Transcriptomics Technology in Cancer Research. Front. Oncol..

[216] Mutuku S.M., Spotbeen X., Trim P.J., Snel M.F., Butler L.M., Swinnen J.V. (2022). Unravelling Prostate Cancer Heterogeneity Using Spatial Approaches to Lipidomics and Transcriptomics. Cancers.

[217] Watanabe R., Miura N., Kurata M., Kitazawa R., Kikugawa T., Saika T. (2023). Spatial Gene Expression Analysis Reveals Characteristic Gene Expression Patterns of De Novo Neuroendocrine Prostate Cancer Coexisting with Androgen Receptor Pathway Prostate Cancer. Int. J. Mol. Sci..

[218] Wang Y., Ma S., Ruzzo W.L. (2020). Spatial Modeling of Prostate Cancer Metabolic Gene Expression Reveals Extensive Heterogeneity and Selective Vulnerabilities. Sci. Rep..

[219] Berglund E., Maaskola J., Schultz N., Friedrich S., Marklund M., Bergenstråhle J., Tarish F., Tanoglidi A., Vickovic S., Larsson L. (2018). Spatial Maps of Prostate Cancer Transcriptomes Reveal an Unexplored Landscape of Heterogeneity. Nat. Commun..

[220] Cunha G.R., Lung B. (1978). The Possible Influence of Temporal Factors in Androgenic Responsiveness of Urogenital Tissue Recombinants from Wild-type and Androgen-insensitive (Tfm) Mice. J. Exp. Zool..

[221] Cunha G.R., Hayward S.W., Wang Y.Z. (2002). Role of Stroma in Carcinogenesis of the Prostate. Differentiation.

[222] Niu Y.N., Xia S.J. (2009). Stroma-Epithelium Crosstalk in Prostate Cancer. Asian J. Androl..

[223] Arnold J.T., Gray N.E., Jacobowitz K., Viswanathan L., Cheung P.W., McFann K.K., Le H., Blackman M.R. (2008). Human Prostate Stromal Cells Stimulate Increased PSA Production in DHEA-Treated Prostate Cancer Epithelial Cells. J. Steroid Biochem. Mol. Biol..

[224] Owen J.S., Clayton A., Pearson H.B. (2023). Cancer-Associated Fibroblast Heterogeneity, Activation and Function: Implications for Prostate Cancer. Biomolecules.

[225] Mo F., Lin D., Takhar M., Ramnarine V.R., Dong X., Bell R.H., Volik S.V., Wang K., Xue H., Wang Y. (2018). Stromal Gene Expression Is Predictive for Metastatic Primary Prostate Cancer. Eur. Urol..

[226] González L.O., Eiro N., Fraile M., Beridze N., Escaf A.R., Escaf S., Fernández-Gómez J.M., Vizoso F.J. (2022). Prostate Cancer Tumor Stroma: Responsibility in Tumor Biology, Diagnosis and Treatment. Cancers.

[227] Henshall S.M., Quinn D.I., Lee C.S., Head D.R., Golovsky D., Brenner P.C., Delprado W., Stricker P.D., Grygiel J.J., Sutherland R.L. (2001). Altered Expression of Androgen Receptor in the Malignant Epithelium and Adjacent Stroma Is Associated with Early Relapse in Prostate Cancer. Cancer Res..

[228] Ricciardelli C., Choong C.S., Buchanan G., Vivekanandan S., Neufing P., Stahl J., Marshall V.R., Horsfall D.J., Tilley W.D. (2005). Androgen Receptor Levels in Prostate Cancer Epithelial and Peritumoral Stromal Cells Identify Non-Organ Confined Disease. Prostate.

[229] Leach D.A., Buchanan G. (2017). Stromal Androgen Receptor in Prostate Cancer Development and Progression. Cancers.

[230] Jia Z., Rahmatpanah F.B., Chen X., Lernhardt W., Wang Y., Xia X.Q., Sawyers A., Sutton M., McClelland M., Mercola D. (2012). Expression Changes in the Stroma of Prostate Cancer Predict Subsequent Relapse. PLoS ONE.

[231] Jia Z., Wang Y., Sawyers A., Yao H., Rahmatpanah F., Xia O.Q., Xu Q., Pio R., Turan T., Koziol J.A. (2011). Diagnosis of Prostate Cancer Using Differentially Expressed Genes in Stroma. Cancer Res..

[232] Kester L., van Oudenaarden A. (2018). Single-Cell Transcriptomics Meets Lineage Tracing. Cell Stem Cell.

[233] Kretzschmar K., Watt F.M. (2012). Lineage Tracing. Cell.

[234] Humphreys B.D., Dirocco D.P. (2014). Lineage-Tracing Methods and the Kidney. Kidney Int..

[235] Klein A.M., Mazutis L., Akartuna I., Tallapragada N., Veres A., Li V., Peshkin L., Weitz D.A., Kirschner M.W. (2015). Droplet Barcoding for Single-Cell Transcriptomics Applied to Embryonic Stem Cells. Cell.

[236] Hsu Y.C. (2015). Theory and Practice of Lineage Tracing. Stem Cells.

[237] Lamprecht S., Schmidt E.M., Blaj C., Hermeking H., Jung A., Kirchner T., Horst D. (2017). Multicolor Lineage Tracing Reveals Clonal Architecture and Dynamics in Colon Cancer. Nat. Commun..

[238] Singh K., Bailey-Lundberg J.M. (2022). Murine Models for Lineage Tracing Cancer Initiating Cells. Methods in Molecular Biology.

[239] Wolf S., Wan Y., McDole K. (2021). Current Approaches to Fate Mapping and Lineage Tracing Using Image Data. Development.

[240] Griffiths J.A., Scialdone A., Marioni J.C. (2018). Using Single-cell Genomics to Understand Developmental Processes and Cell Fate Decisions. Mol. Syst. Biol..

[241] VanHorn S., Morris S.A. (2021). Next-Generation Lineage Tracing and Fate Mapping to Interrogate Development. Dev. Cell.

[242] Forrow A., Schiebinger G. (2021). LineageOT Is a Unified Framework for Lineage Tracing and Trajectory Inference. Nat. Commun..

[243] Wagner D.E., Klein A.M. (2020). Lineage Tracing Meets Single-Cell Omics: Opportunities and Challenges. Nat. Rev. Genet..

[244] Fletcher R.B., Das D., Ngai J. (2018). Creating Lineage Trajectory Maps Via Integration of Single-Cell RNA-Sequencing and Lineage Tracing. BioEssays.

[245] Chen C., Liao Y., Peng G. (2022). Connecting Past and Present: Single-Cell Lineage Tracing. Protein Cell.

[246] Gabbutt C., Schenck R.O., Weisenberger D.J., Kimberley C., Berner A., Househam J., Lakatos E., Robertson-Tessi M., Martin I., Patel R. (2022). Fluctuating Methylation Clocks for Cell Lineage Tracing at High Temporal Resolution in Human Tissues. Nat. Biotechnol..

[247] Marjanovic N.D., Hofree M., Chan J.E., Canner D., Wu K., Trakala M., Hartmann G.G., Smith O.C., Kim J.Y., Evans K.V. (2020). Emergence of a High-Plasticity Cell State during Lung Cancer Evolution. Cancer Cell.

[248] LaFave L.M., Kartha V.K., Ma S., Meli K., Del Priore I., Lareau C., Naranjo S., Westcott P.M.K., Duarte F.M., Sankar V. (2020). Epigenomic State Transitions Characterize Tumor Progression in Mouse Lung Adenocarcinoma. Cancer Cell.

[249] Blanco M.A., Sykes D.B., Gu L., Wu M., Petroni R., Karnik R., Wawer M., Rico J., Li H., Jacobus W.D. (2021). Chromatin-State Barriers Enforce an Irreversible Mammalian Cell Fate Decision. Cell Rep..

[250] Berenguer J., Celià-Terrassa T. (2021). Cell Memory of Epithelial-Mesenchymal Plasticity in Cancer. Curr. Opin. Cell Biol..

[251] Tang F., Xu D., Wang S., Wong C.K., Martinez-Fundichely A., Lee C.J., Cohen S., Park J., Hill C.E., Eng K. (2022). Chromatin Profiles Classify Castration-Resistant Prostate Cancers Suggesting Therapeutic Targets. Science.

[252] Soundararajan R., Viscuse P., Pilie P., Liu J., Logotheti S., Laberiano Fernández C., Lorenzini D., Hoang A., Lu W., Soto L.M. (2022). Genotype-to-Phenotype Associations in the Aggressive Variant Prostate Cancer Molecular Profile (AVPC-m) Components. Cancers.

[253] Formaggio N., Rubin M.A., Theurillat J.P. (2021). Loss and Revival of Androgen Receptor Signaling in Advanced Prostate Cancer. Oncogene.

[254] Ling B., Liao X., Huang Y., Liang L., Jiang Y., Pang Y., Qi G. (2020). Identification of Prognostic Markers of Lung Cancer through Bioinformatics Analysis and in Vitro Experiments. Int. J. Oncol..

[255] Huang G.J., Yang B.B. (2021). Identification of Core MiRNA Prognostic Markers in Patients with Laryngeal Cancer Using Bioinformatics Analysis. Eur. Arch. Oto-Rhino-Laryngol..

[256] Chen Y., Gong Y., Dou L., Zhou X., Zhang Y. (2022). Bioinformatics Analysis Methods for Cell-Free DNA. Comput. Biol. Med..

[257] Gao Y., Liu J., Qian X., He X. (2021). Identification of Markers Associated with Brain Metastasis from Breast Cancer through Bioinformatics Analysis and Verification in Clinical Samples. Gland Surg..

[258] Huang H., Zhang Q., Ye C., Lv J.M., Liu X., Chen L., Wu H., Yin L., Cui X.G., Xu D.F. (2017). Identification of Prognostic Markers of High Grade Prostate Cancer through an Integrated Bioinformatics Approach. J. Cancer Res. Clin. Oncol..

[259] Ye D.M., Xu G., Ma W., Li Y., Luo W., Xiao Y., Liu Y., Zhang Z. (2020). Significant Function and Research Progress of Biomarkers in Gastric Cancer (Review). Oncol. Lett..

[260] Van De Vijver M.J. (2014). Molecular Tests as Prognostic Factors in Breast Cancer. Virchows Arch..

[261] Hynst J., Navrkalova V., Pal K., Pospisilova S. (2021). Bioinformatic Strategies for the Analysis of Genomic Aberrations Detected by Targeted NGS Panels with Clinical Application. PeerJ.

[262] Garcia-Moreno A., López-Domínguez R., Villatoro-García J.A., Ramirez-Mena A., Aparicio-Puerta E., Hackenberg M., Pascual-Montano A., Carmona-Saez P. (2022). Functional Enrichment Analysis of Regulatory Elements. Biomedicines.

[263] Zhao K., Rhee S.Y. (2023). Interpreting Omics Data with Pathway Enrichment Analysis. Trends Genet..

[264] Cibulskis K., Lawrence M.S., Carter S.L., Sivachenko A., Jaffe D., Sougnez C., Gabriel S., Meyerson M., Lander E.S., Getz G. (2013). Sensitive Detection of Somatic Point Mutations in Impure and Heterogeneous Cancer Samples. Nat. Biotechnol..

[265] do Valle Í.F., Giampieri E., Simonetti G., Padella A., Manfrini M., Ferrari A., Papayannidis C., Zironi I., Garonzi M., Bernardi S. (2016). Optimized Pipeline of MuTect and GATK Tools to Improve the Detection of Somatic Single Nucleotide Polymorphisms in Whole-Exome Sequencing Data. BMC Bioinform..

[266] Cibulskis K., Lawrence M.S., Carter S.L., Sivachenko A., Jaffe D., Sougnez C., Gabriel S., Meyerson M., Lander E.S., Getz G. (2013). MuTect—Brief Summary. Nat. Biotechnol..

[267] Mayakonda A., Lin D.C., Assenov Y., Plass C., Koeffler H.P. (2018). Maftools: Efficient and Comprehensive Analysis of Somatic Variants in Cancer. Genome Res..

[268] Wen C., Ge Q., Dai B., Li J., Yang F., Meng J., Gao S., Fan S., Zhang L. (2022). Signature for Prostate Cancer Based on Autophagy-Related Genes and a Nomogram for Quantitative Risk Stratification. Dis. Markers.

[269] Minussi D.C., Sei E., Wang J., Schalck A., Yan Y., Davis A., Wu H.-J., Bai S., Peng C., Hu M. (2022). Resolving Clonal Substructure from Single Cell Genomic Data Using CopyKit. bioRxiv.

[270] Mallory X.F., Edrisi M., Navin N., Nakhleh L. (2020). Assessing the Performance of Methods for Copy Number Aberration Detection from Single-Cell DNA Sequencing Data. PLoS Comput. Biol..

[271] Zaccaria S., Raphael B.J. (2021). Characterizing the Allele- and Haplotype-Specific Copy Number Landscape of Cancer Genomes at Single-Cell Resolution with CHISEL. Nat. Biotechnol..

[272] Garvin T., Aboukhalil R., Kendall J., Baslan T., Atwal G.S., Hicks J., Wigler M., Schatz M.C. (2015). Interactive Analysis and Assessment of Single-Cell Copy-Number Variations. Nat. Methods.

[273] Schiebinger G., Shu J., Tabaka M., Cleary B., Subramanian V., Solomon A., Gould J., Liu S., Lin S., Berube P. (2019). Optimal-Transport Analysis of Single-Cell Gene Expression Identifies Developmental Trajectories in Reprogramming. Cell.

[274] Qiu X., Mao Q., Tang Y., Wang L., Chawla R., Pliner H.A., Trapnell C. (2017). Reversed Graph Embedding Resolves Complex Single-Cell Trajectories. Nat. Methods.

[275] Trapnell C., Cacchiarelli D., Grimsby J., Pokharel P., Li S., Morse M., Lennon N.J., Livak K.J., Mikkelsen T.S., Rinn J.L. (2014). The Dynamics and Regulators of Cell Fate Decisions Are Revealed by Pseudotemporal Ordering of Single Cells. Nat. Biotechnol..

[276] Satija R., Farrell J.A., Gennert D., Schier A.F., Regev A. (2015). Spatial Reconstruction of Single-Cell Gene Expression Data. Nat. Biotechnol..

[277] Butler A., Hoffman P., Smibert P., Papalexi E., Satija R. (2018). Integrating Single-Cell Transcriptomic Data across Different Conditions, Technologies, and Species. Nat. Biotechnol..

[278] Tirosh I., Izar B., Prakadan S.M., Wadsworth M.H., Treacy D., Trombetta J.J., Rotem A., Rodman C., Lian C., Murphy G. (2016). Dissecting the Multicellular Ecosystem of Metastatic Melanoma by Single-Cell RNA-Seq. Science.

[279] Andreatta M., Carmona S.J. (2021). UCell: Robust and Scalable Single-Cell Gene Signature Scoring. Comput. Struct. Biotechnol. J..

[280] Stuart T., Butler A., Hoffman P., Hafemeister C., Papalexi E., Mauck W.M., Hao Y., Stoeckius M., Smibert P., Satija R. (2019). Comprehensive Integration of Single-Cell Data. Cell.

[281] Gulati G.S., Sikandar S.S., Wesche D.J., Manjunath A., Bharadwaj A., Berger M.J., Ilagan F., Kuo A.H., Hsieh R.W., Cai S. (2020). Single-Cell Transcriptional Diversity Is a Hallmark of Developmental Potential. Science.

[282] Zhang Y., Liu T., Meyer C.A., Eeckhoute J., Johnson D.S., Bernstein B.E., Nussbaum C., Myers R.M., Brown M., Li W. (2008). Model-Based Analysis of ChIP-Seq (MACS). Genome Biol..

[283] Feng J., Liu T., Qin B., Zhang Y., Liu X.S. (2012). Identifying ChIP-Seq Enrichment Using MACS. Nat. Protoc..

[284] Gaspar J.M. (2018). Improved Peak-Calling with MACS2. bioRxiv.

[285] Xu S., Grullon S., Ge K., Peng W. (2014). Spatial Clustering for Identification of Chip-Enriched Regions (SICER) to Map Regions of Histone Methylation Patterns in Embryonic Stem Cells. Methods Mol. Biol..

[286] Yu G., Wang L.G., He Q.Y. (2015). ChIP Seeker: An R/Bioconductor Package for ChIP Peak Annotation, Comparison and Visualization. Bioinformatics.

[287] Krueger F., Andrews S.R. (2011). Bismark: A Flexible Aligner and Methylation Caller for Bisulfite-Seq Applications. Bioinformatics.

[288] Chen P.-Y., Cokus S.J., Pellegrini M. (2010). BS Seeker: Precise Mapping for Bisulfite Sequencing. BMC Bioinform..

[289] Akalin A., Kormaksson M., Li S., Garrett-Bakelman F.E., Figueroa M.E., Melnick A., Mason C.E. (2012). MethylKit: A Comprehensive R Package for the Analysis of Genome-Wide DNA Methylation Profiles. Genome Biol..

[290] Akalin A., Franke V., Vlahoviček K., Mason C.E., Schübeler D. (2015). Genomation: A Toolkit to Summarize, Annotate and Visualize Genomic Intervals. Bioinformatics.

[291] Fang R., Preissl S., Li Y., Hou X., Lucero J., Wang X., Motamedi A., Shiau A.K., Zhou X., Xie F. (2021). Comprehensive Analysis of Single Cell ATAC-Seq Data with SnapATAC. Nat. Commun..

[292] Ma W., Lu J., Wu H. (2023). Cellcano: Supervised Cell Type Identification for Single Cell ATAC-Seq Data. Nat. Commun..

[293] Stuart T., Srivastava A., Madad S., Lareau C.A., Satija R. (2021). Single-Cell Chromatin State Analysis with Signac. Nat. Methods.

[294] Chen X., Chen S., Song S., Gao Z., Hou L., Zhang X., Lv H., Jiang R. (2022). Cell Type Annotation of Single-Cell Chromatin Accessibility Data via Supervised Bayesian Embedding. Nat. Mach. Intell..

[295] Subramanian A., Tamayo P., Mootha V.K., Mukherjee S., Ebert B.L., Gillette M.A., Paulovich A., Pomeroy S.L., Golub T.R., Lander E.S. (2005). Gene Set Enrichment Analysis: A Knowledge-Based Approach for Interpreting Genome-Wide Expression Profiles. Proc. Natl. Acad. Sci. USA.

[296] Krämer A., Green J., Pollard J., Tugendreich S. (2014). Causal Analysis Approaches in Ingenuity Pathway Analysis. Bioinformatics.

[297] Chen E.Y., Tan C.M., Kou Y., Duan Q., Wang Z., Meirelles G.V., Clark N.R., Ma’ayan A. (2013). Enrichr: Interactive and Collaborative HTML5 Gene List Enrichment Analysis Tool. BMC Bioinform..

[298] Kuleshov M.V., Jones M.R., Rouillard A.D., Fernandez N.F., Duan Q., Wang Z., Koplev S., Jenkins S.L., Jagodnik K.M., Lachmann A. (2016). Enrichr: A Comprehensive Gene Set Enrichment Analysis Web Server 2016 Update. Nucleic Acids Res..

[299] Xie Z., Bailey A., Kuleshov M.V., Clarke D.J.B., Evangelista J.E., Jenkins S.L., Lachmann A., Wojciechowicz M.L., Kropiwnicki E., Jagodnik K.M. (2021). Gene Set Knowledge Discovery with Enrichr. Curr. Protoc..

[300] Thanati F., Karatzas E., Baltoumas F.A., Stravopodis D.J., Eliopoulos A.G., Pavlopoulos G.A. (2021). FLAME: A Web Tool for Functional and Literature Enrichment Analysis of Multiple Gene Lists. Biology.

[301] Shah S.P., Xuan X., DeLeeuw R.J., Khojasteh M., Lam W.L., Ng R., Murphy K.P. (2006). Integrating Copy Number Polymorphisms into Array CGH Analysis Using a Robust HMM. Bioinformatics.

[302] Mallory X.F., Edrisi M., Navin N., Nakhleh L. (2020). Methods for Copy Number Aberration Detection from Single-Cell DNA-Sequencing Data. Genome Biol..

[303] Berger M.F., Lawrence M.S., Demichelis F., Drier Y., Cibulskis K., Sivachenko A.Y., Sboner A., Esgueva R., Pflueger D., Sougnez C. (2011). The Genomic Complexity of Primary Human Prostate Cancer. Nature.

[304] Jaratlerdsiri W., Chan E.K.F., Gong T., Petersen D.C., Kalsbeek A.M.F., Venter P.A., Stricker P.D., Riana Bornman M.S., Hayes V.M. (2018). Whole-Genome Sequencing Reveals Elevated Tumor Mutational Burden and Initiating Driver Mutations in African Men with Treatment-Naïve, High-Risk Prostate Cancer. Cancer Res..

[305] Hong M.K.H., Macintyre G., Wedge D.C., Van Loo P., Patel K., Lunke S., Alexandrov L.B., Sloggett C., Cmero M., Marass F. (2015). Tracking the Origins and Drivers of Subclonal Metastatic Expansion in Prostate Cancer. Nat. Commun..

[306] Rajendran B.K., Deng C.X. (2017). A Comprehensive Genomic Meta-Analysis Identifies Confirmatory Role of OBSCN Gene in Breast Tumorigenesis. Oncotarget.

[307] Tolkach Y., Kristiansen G. (2018). The Heterogeneity of Prostate Cancer: A Practical Approach. Pathobiology.

[308] Mateo J., Seed G., Bertan C., Rescigno P., Dolling D., Figueiredo I., Miranda S., Rodrigues D.N., Gurel B., Clarke M. (2020). Genomics of Lethal Prostate Cancer at Diagnosis and Castration Resistance. J. Clin. Investig..

[309] Sumanasuriya S., Seed G., Parr H., Christova R., Pope L., Bertan C., Bianchini D., Rescigno P., Figueiredo I., Goodall J. (2021). Elucidating Prostate Cancer Behaviour During Treatment via Low-Pass Whole-Genome Sequencing of Circulating Tumour DNA. Eur. Urol..

[310] Choudhury A.D., Werner L., Francini E., Wei X.X., Ha G., Freeman S.S., Rhoades J., Reed S.C., Gydush G., Rotem D. (2018). Tumor Fraction in Cell-Free DNA as a Biomarker in Prostate Cancer. JCI Insight.

[311] Rodriguez A., Laio A. (2014). Machine Learning. Clustering by Fast Search and Find of Density Peaks. Science.

[312] Mao Q., Wang L., Goodison S., Sun Y. Dimensionality Reduction via Graph Structure Learning. Proceedings of the ACM SIGKDD International Conference on Knowledge Discovery and Data Mining.

[313] Mao Q., Wang L., Tsang I.W., Sun Y. (2017). Principal Graph and Structure Learning Based on Reversed Graph Embedding. IEEE Trans. Pattern Anal. Mach. Intell..

[314] Qiu X., Hill A., Packer J., Lin D., Ma Y.A., Trapnell C. (2017). Single-Cell MRNA Quantification and Differential Analysis with Census. Nat. Methods.

[315] Risbridger G.P., Clark A.K., Porter L.H., Toivanen R., Bakshi A., Lister N.L., Pook D., Pezaro C.J., Sandhu S., Keerthikumar S. (2021). The MURAL Collection of Prostate Cancer Patient-Derived Xenografts Enables Discovery through Preclinical Models of Uro-Oncology. Nat. Commun..

[316] Dong B., Miao J., Wang Y., Luo W., Ji Z., Lai H., Zhang M., Cheng X., Wang J., Fang Y. (2020). Single-Cell Analysis Supports a Luminal-Neuroendocrine Transdifferentiation in Human Prostate Cancer. Commun. Biol..

[317] Baures M., Puig Lombardi E., Di Martino D., Zeitouni W., Pacreau E., Dos Santos L., Dariane C., Boutillon F., Guidotti J.E., Goffin V. (2022). Transcriptomic Signature and Growth Factor Regulation of Castration-Tolerant Prostate Luminal Progenitor Cells. Cancers.

[318] Valdés-Mora F., Clark S.J. (2015). Prostate Cancer Epigenetic Biomarkers: Next-Generation Technologies. Oncogene.

[319] Tonmoy M.I.Q., Fariha A., Hami I., Kar K., Reza H.A., Bahadur N.M., Hossain M.S. (2022). Computational Epigenetic Landscape Analysis Reveals Association of CACNA1G-AS1, F11-AS1, NNT-AS1, and MSC-AS1 LncRNAs in Prostate Cancer Progression through Aberrant Methylation. Sci. Rep..

[320] Zhang C., Macchi F., Magnani E., Sadler K.C. (2021). Chromatin States Shaped by an Epigenetic Code Confer Regenerative Potential to the Mouse Liver. Nat. Commun..

[321] He Y., Wei T., Ye Z., Orme J.J., Lin D., Sheng H., Fazli L., Jeffrey Karnes R., Jimenez R., Wang L. (2021). A Noncanonical AR Addiction Drives Enzalutamide Resistance in Prostate Cancer. Nat. Commun..

[322] Cejas P., Xie Y., Font-Tello A., Lim K., Syamala S., Qiu X., Tewari A.K., Shah N., Nguyen H.M., Patel R.A. (2021). Subtype Heterogeneity and Epigenetic Convergence in Neuroendocrine Prostate Cancer. Nat. Commun..

[323] Stelloo S., Nevedomskaya E., Kim Y., Schuurman K., Valle-Encinas E., Lobo J., Krijgsman O., Peeper D.S., Chang S.L., Feng F.Y.C. (2018). Integrative Epigenetic Taxonomy of Primary Prostate Cancer. Nat. Commun..

[324] Zang C., Schones D.E., Zeng C., Cui K., Zhao K., Peng W. (2009). A Clustering Approach for Identification of Enriched Domains from Histone Modification ChIP-Seq Data. Bioinformatics.

[325] Coleman D.J., Gao L., Schwartzman J., Korkola J.E., Sampson D., Derrick D.S., Urrutia J., Balter A., Burchard J., King C.J. (2019). Maintenance of MYC Expression Promotes de Novo Resistance to BET Bromodomain Inhibition in Castration-Resistant Prostate Cancer. Sci. Rep..

[326] Dhar S., Kumar A., Gomez C.R., Akhtar I., Hancock J.C., Lage J.M., Pound C.R., Levenson A.S. (2017). MTA1-Activated Epi-MicroRNA-22 Regulates E-Cadherin and Prostate Cancer Invasiveness. FEBS Lett..

[327] Xiao L., Parolia A., Qiao Y., Bawa P., Eyunni S., Mannan R., Carson S.E., Chang Y., Wang X., Zhang Y. (2022). Targeting SWI/SNF ATPases in Enhancer-Addicted Prostate Cancer. Nature.

[328] Cranor L.F. (1994). Programming Perl. XRDS Crossroads ACM Mag. Stud..

[329] Rothwell W.B. (2020). Advanced Perl Programming.

[330] Langmead B., Trapnell C., Pop M., Salzberg S.L. (2009). Ultrafast and Memory-Efficient Alignment of Short DNA Sequences to the Human Genome. Genome Biol..

[331] B Langmead C.T.M.P.S.S. (2009). Bowtie: An Ultrafast Memory-Efficient Short Read Aligner. Genome Biol..

[332] Langmead B., Salzberg S. (2013). Fast gapped-read alignment with Bowtie2. Nat. Methods.

[333] Kipfer B.A. (2021). Bowtie. Encyclopedic Dictionary of Archaeology.

[334] Zhao S.G., Chen W.S., Li H., Foye A., Zhang M., Sjöström M., Aggarwal R., Playdle D., Liao A., Alumkal J.J. (2020). The DNA Methylation Landscape of Advanced Prostate Cancer. Nat. Genet..

[335] Yu Y.P., Ding Y., Chen R., Liao S.G., Ren B.G., Michalopoulos A., Michalopoulos G., Nelson J., Tseng G.C., Luo J.H. (2013). Whole-Genome Methylation Sequencing Reveals Distinct Impact of Differential Methylations on Gene Transcription in Prostate Cancer. Am. J. Pathol..

[336] Li J., Xu C., Lee H.J., Ren S., Zi X., Zhang Z., Wang H., Yu Y., Yang C., Gao X. (2020). A Genomic and Epigenomic Atlas of Prostate Cancer in Asian Populations. Nature.

[337] Schmidt T., Leha A., Salinas-Riester G. (2016). Treatment of Prostate Cancer Cells with S-Adenosylmethionine Leads to Genome-Wide Alterations in Transcription Profiles. Gene.

[338] Ketola K., Kaljunen H., Taavitsainen S., Kaarijärvi R., Järvelä E., Rodríguez-Martín B., Haase K., Woodcock D.J., Tubio J., Wedge D.C. (2021). Subclone Eradication Analysis Identifies Targets for Enhanced Cancer Therapy and Reveals L1 Retrotransposition as a Dynamic Source of Cancer Heterogeneity. Cancer Res..

[339] Eksi S.E., Chitsazan A., Sayar Z., Thomas G.V., Fields A.J., Kopp R.P., Spellman P.T., Adey A.C. (2021). Epigenetic Loss of Heterogeneity from Low to High Grade Localized Prostate Tumours. Nat. Commun..

[340] Liu Y., Shen S., Lapata M. Noisy Self-Knowledge Distillation for Text Summarization. Proceedings of the NAACL-HLT 2021-2021 Conference of the North American Chapter of the Association for Computational Linguistics: Human Language Technologies.

[341] Kneppers J., Severson T.M., Siefert J.C., Schol P., Joosten S.E.P., Yu I.P.L., Huang C.C.F., Morova T., Altıntaş U.B., Giambartolomei C. (2022). Extensive Androgen Receptor Enhancer Heterogeneity in Primary Prostate Cancers Underlies Transcriptional Diversity and Metastatic Potential. Nat. Commun..

[342] Taavitsainen S., Engedal N., Cao S., Handle F., Erickson A., Prekovic S., Wetterskog D., Tolonen T., Vuorinen E.M., Kiviaho A. (2021). Single-Cell ATAC and RNA Sequencing Reveal Pre-Existing and Persistent Cells Associated with Prostate Cancer Relapse. Nat. Commun..

[343] Weinstein J.N., Collisson E.A., Mills G.B., Shaw K.R.M., Ozenberger B.A., Ellrott K., Sander C., Stuart J.M., Chang K., Creighton C.J. (2013). The Cancer Genome Atlas Pan-Cancer Analysis Project. Nat. Genet..

[344] Edgar R., Domrachev M., Lash A.E. (2002). Gene Expression Omnibus: NCBI Gene Expression and Hybridization Array Data Repository. Nucleic Acids Res..

[345] Hung J.-H., Yang T.-H., Hu Z., Weng Z., DeLisi C. (2012). Gene Set Enrichment Analysis: Performance Evaluation and Usage Guidelines. Brief. Bioinform..

[346] Zhao Y., Tao Z., Li L., Zheng J., Chen X. (2022). Predicting Biochemical-Recurrence-Free Survival Using a Three-Metabolic-Gene Risk Score Model in Prostate Cancer Patients. BMC Cancer.

[347] Wang Y., Wang J., Tang Q., Ren G. (2021). Identification of UBE2C as Hub Gene in Driving Prostate Cancer by Integrated Bioinformatics Analysis. PLoS ONE.

[348] Yu C., Cao H., He X., Sun P., Feng Y., Chen L., Gong H. (2017). Cyclin-Dependent Kinase Inhibitor 3 (CDKN3) Plays a Critical Role in Prostate Cancer via Regulating Cell Cycle and DNA Replication Signaling. Biomed. Pharmacother..

[349] Chen W.S., Aggarwal R., Zhang L., Zhao S.G., Thomas G.V., Beer T.M., Quigley D.A., Foye A., Playdle D., Huang J. (2019). Genomic Drivers of Poor Prognosis and Enzalutamide Resistance in Metastatic Castration-Resistant Prostate Cancer (Figure Presented.). Eur. Urol..

[350] Gong H., Chen X.Y., Jin Y.C., Lu J.S., Cai Y.J., Wei O., Zhao J., Zhang W.Y., Wen X.F., Wang Y.M. (2019). Expression of ARHGAP10 Correlates with Prognosis of Prostate Cancer. Int. J. Clin. Exp. Pathol..

[351] Patel R., Brzezinska E.A., Repiscak P., Ahmad I., Mui E., Gao M., Blomme A., Harle V., Tan E.H., Malviya G. (2020). Activation of β-Catenin Cooperates with Loss of Pten to Drive AR-Independent Castration-Resistant Prostate Cancer. Cancer Res..

[352] Irshad S., Bansal M., Castillo-Martin M., Zheng T., Aytes A., Wenske S., Le Magnen C., Guarnieri P., Sumazin P., Benson M.C. (2013). A Molecular Signature Predictive of Indolent Prostate Cancer. Sci. Transl. Med..

[353] Chen W.Y., Wen Y.C., Lin S.R., Yeh H.L., Jiang K.C., Chen W.H., Lin Y.S., Zhang Q., Liew P.L., Hsiao M. (2021). Nerve Growth Factor Interacts with CHRM4 and Promotes Neuroendocrine Differentiation of Prostate Cancer and Castration Resistance. Commun. Biol..

[354] Savli H., Szendröi A., Romics I., Nagy B. (2008). Gene Network and Canonical Pathway Analysis in Prostate Cancer: A Microarray Study. Exp. Mol. Med..

[355] Sethi S., Kong D., Land S., Dyson G., Sakr W.A., Sarkar F.H. (2013). Comprehensive Molecular Oncogenomic Profiling and MiRNA Analysis of Prostate Cancer. Am. J. Transl. Res..

[356] Wu X.C., Yu Y.Z., Zuo Y.Z., Song X.L., Zhou Z.E., Xiao Y., Luo D.S., Yan W.G., Zhao S.C. (2022). Identification of UAP1L1 as a Critical Factor for Prostate Cancer and Underlying Molecular Mechanism in Tumorigenicity. J. Transl. Med..

[357] Russo A.L., Jedlicka K., Wernick M., McNally D., Kirk M., Sproull M., Smith S., Shankavaram U., Kaushal A., Figg W.D. (2009). Urine Analysis and Protein Networking Identify Met as a Marker of Metastatic Prostate Cancer. Clin. Cancer Res..

[358] Farashi S., Kryza T., Batra J. (2020). Pathway Analysis of Genes Identified through Post-GWAS to Underpin Prostate Cancer Aetiology. Genes.

[359] Nagaya N., Rosenfeld J., Lee G.T., Kim I.Y. (2021). RNA-Seq Profile of African American Men with a Clinically Localized Prostate Cancer. Prostate Int..

[360] Szklarczyk D., Franceschini A., Wyder S., Forslund K., Heller D., Huerta-Cepas J., Simonovic M., Roth A., Santos A., Tsafou K.P. (2015). STRING V10: Protein-Protein Interaction Networks, Integrated over the Tree of Life. Nucleic Acids Res..

[361] Woodworth M.B., Girskis K.M., Walsh C.A. (2017). Building a Lineage from Single Cells: Genetic Techniques for Cell Lineage Tracking. Nat. Rev. Genet..

[362] Castro L.N.G., Tirosh I., Suvà M.L. (2021). Decoding Cancer Biology One Cell at a Time. Cancer Discov..

[363] LaFave L.M., Savage R.E., Buenrostro J.D. (2022). Single-Cell Epigenomics Reveals Mechanisms of Cancer Progression. Annu. Rev. Cancer Biol..

[364] Guruprasad P., Lee Y.G., Kim K.H., Ruella M. (2021). The Current Landscape of Single-Cell Transcriptomics for Cancer Immunotherapy. J. Exp. Med..

[365] Bernard V., Semaan A., Huang J., Anthony San Lucas F., Mulu F.C., Stephens B.M., Guerrero P.A., Huang Y., Zhao J., Kamyabi N. (2019). Single-Cell Transcriptomics of Pancreatic Cancer Precursors Demonstrates Epithelial and Microenvironmental Heterogeneity as an Early Event in Neoplastic Progression. Clin. Cancer Res..

[366] Wu S.Z., Al-Eryani G., Roden D.L., Junankar S., Harvey K., Andersson A., Thennavan A., Wang C., Torpy J.R., Bartonicek N. (2021). A Single-Cell and Spatially Resolved Atlas of Human Breast Cancers. Nat. Genet..

[367] Saviano A., Henderson N.C., Baumert T.F. (2020). Single-Cell Genomics and Spatial Transcriptomics: Discovery of Novel Cell States and Cellular Interactions in Liver Physiology and Disease Biology. J. Hepatol..

[368] Siefert J.C., Cioni B., Muraro M.J., Alshalalfa M., Vivie J., van der Poel H.G., Schoots I.G., Bekers E., Feng F.Y., Wessels L.F.A. (2021). The Prognostic Potential of Human Prostate Cancer-Associated Macrophage Subtypes as Revealed by Single-Cell Transcriptomics. Mol. Cancer Res..

[369] Shendure J., Balasubramanian S., Church G.M., Gilbert W., Rogers J., Schloss J.A., Waterston R.H. (2017). DNA Sequencing at 40: Past, Present and Future. Nature.

[370] Baylin S.B., Jones P.A. (2016). Epigenetic Determinants of Cancer. Cold Spring Harb. Perspect. Biol..

